# Domestic pigs are susceptible to experimental infection with non-human primate-derived Reston virus without the need for adaptation

**DOI:** 10.1038/s41598-024-51280-8

**Published:** 2024-01-06

**Authors:** Charles E. Lewis, Mathieu M. Pinette, Steven M. Lakin, Greg Smith, Mathew Fisher, Estella Moffat, Carissa Embury-Hyatt, Brad S. Pickering

**Affiliations:** 1 Department of Veterinary Microbiology and Preventive Medicine, College of Veterinary Medicine, Iowa State University, Ames, IA USA; 2 Interdepartmental Microbiology Program, College of Agriculture and Life Sciences, Iowa State University, Ames, IA USA; 3 National Centre for Foreign Animal Disease, Canadian Food Inspection Agency, Winnipeg, MB Canada; 4 Scientific Liaison Services Section, Foreign Animal Disease Diagnostic Laboratory, National Veterinary Services Laboratories, Animal Plant Health Inspection Service, United States Department of Agriculture, Orient Point, NY USA; 5 Department of Medical Microbiology and Infectious Diseases, University of Manitoba, Winnipeg, MB Canada

**Keywords:** Pathogens, Virology

## Abstract

Domestic pigs are a critical component of the food supply and one of the most commonly raised production animals. Pork consumption has driven the intensification of pig production expanding into environments conducive to increased emergence and spread of infectious diseases, including the spillover of pathogens into human populations. One of these emerging viruses, Reston virus (RESTV), is an enigma among the *Orthoebolavirus* genus in that its lack of human pathogenicity is in stark contrast to the high virulence associated with most other ebolaviruses. RESTV is, however, associated with outbreaks of highly lethal hemorrhagic disease in non-human primates (NHP), as well as poorly understood clinical manifestations of mixed virulence and lethality in naturally and experimentally infected domestic pigs. Our results show it is possible for RESTV derived from an NHP to infect domestic pigs resulting in a spectrum of disease, from asymptomatic to severe respiratory distress. Further, we report on the first experimental transmission of RESTV between infected pigs and a co-housed, naïve animal, as well as the first report of the successful use of group oral fluids for the detection of RESTV RNA and virus-specific IgA antibodies.

## Introduction

Reston virus (RESTV) is a negative sense, non-segmented RNA virus of the family *Filoviridae*, genus *Orthoebolavirus*, that was discovered in 1989 during an outbreak in non-human primate (NHP) quarantine facilities in Virginia and Pennsylvania, USA, involving cynomolgus macaques (*Macaca fascicularis*) imported from the Philippines^1,2^. Since that time, sporadic outbreaks have been reported surrounding repeat incidents in the United States and Italy, all traceable to the Island of Luzon, Philippines^3^. In 2008, RESTV emerged in domestic pigs (*Sus scrofa domesticus*) from the Island of Luzon, and, subsequently, ebolavirus sequences and virus-specific antibodies have been detected in pigs from a variety of locations, including China, Guinea, Sierra Leone, and Uganda^4–8^. The presence of RESTV and other ebolaviruses in the human food chain substantiates the relationship with domestic pigs with these pathogens as a public health concern requiring further investigation^4,9,10^.

RESTV is a zoonotic pathogen, known to be capable of infecting humans. In contrast to the other *Orthoebolavirus*, though, infection has not been associated with the development of overt, appreciable disease; and the reasons for this lack of pathogenicity are unknown^11–13^. Numerous human exposures have been suggested by the detection of antibodies in persons with close contact to either infected NHPs or pigs^9,13^. Furthermore, infectious virus has been successfully recovered from a single human case that became viremic after an injury sustained during the necropsy of an NHP infected with RESTV^9,11^. Since 2008, another member of the genus, Ebola virus (EBOV), has also been shown to not only be capable of experimentally infecting pigs, but also capable of transmission between pigs and from infected pigs to co-housed NHPs through indirect contact that ultimately resulted in lethal disease^14,15^. The association of an ebolavirus with an intensively produced livestock animal is especially concerning considering the ability of RESTV to asymptomatically infect humans and pigs allowing for the possibility of repeated re-introduction and replication in both populations increasing the risk for changes in virulence^4,10,16^.

Even with forty-six years of extensive research focused on understanding the unusually high virulence typical of most of the ebolaviruses, pathogen- and host-specific factors that contribute to the absence or development of disease are poorly understood^3,12^. This lack of knowledge coupled with the requirement for maximum containment facilities to manipulate these viruses has limited the scientific understanding of ebolavirus biology and pathogenesis, hampering the development of effective mitigation strategies. Considered the gold standard animal model for studying ebolaviruses, the NHP model has been a critical tool for both understanding virus pathogenesis and for evaluating the efficacy of novel countermeasures^17,18^. Much of this utility is owed to the susceptibility of cynomolgus and rhesus macaques to wild-type isolates; but use of these species is accompanied by several disadvantages, including high costs, limited availability, and increased ethical concerns^18^. More recently, the ferret model has gained some traction with its ability to recapitulate consistent hallmarks of human disease, its uniform lethality, and its susceptibility to wild-type ebolavirus isolates^17^.

The vast majority of research concerning the pathogenicity of RESTV has been focused on counter-comparison to EBOV; and almost all previous animal studies in NHPs used the isolate Reston virus/M.fasicularis-tc/USA/1989/Philippines89-Pennsylvania (GenBank accession NC_004161), a virus derived from a cynomolgus macaque involved in the 1989 epizootic^11,17^. Studies focused on RESTV have been predominately limited to NHPs or ferrets, as those models are the only currently available, immunocompetent animal models for regular use in laboratory investigations using wild-type RESTV. The development of other laboratory rodent models have required either the development of a species-adapted isolate through repeated animal passaging of the virus or required the use of transgenic, immunomodulated animals, like STAT-1 knockout mice^17,19^. Therefore, the susceptibility of domestic pigs to wild-type virus may prove useful for scientific investigations into spillover dynamics in a naturally susceptible species.

Similar to what has been documented in human ebolavirus outbreaks, epizootics in pigs likely result from direct spillover events from the reservoir species^8^. For the pigs in the Philippines, this is thought to have occurred as at least three independent introductions, each resulting in sustained pig-to-pig transmission for an unknown amount of time^4^. In particular, these transmission chains are worrisome as they have the potential to provide increased opportunity for host adaptation of the virus^4,9,11^. Although domestic pigs have been shown to be a host for ebolaviruses, limited work has been performed to identify the role suids may play in regards to future outbreaks in humans. Understanding the host–pathogen relationship between swine and RESTV has been largely neglected with studies limited to the use of isolates derived from a naturally infected pig after sustained intra-species transmission during the 2008 epizootic^4,10,20^. Previous RESTV infection studies with domestic pigs have also resulted in conflicting results with discrepancies concerning the development of clinically apparent disease and subsequent pathology. This disparity in results has been postulated to be due to a number of variables, including the effect of inoculum dose, pig breed and genetics, or co-infection with unrelated pathogens^10,20^.

In 2011, a series of experiments were conducted in which five- to six-week-old pigs were inoculated with 10^6^ TCID_50_, either oronasally or subcutaneously, of an isolate of RESTV originating from a naturally infected pig^20^. All pigs in this study, regardless of inoculation method, had virus detected in multiple organs indicating varying levels of systemic spread. The oronasally inoculated animals were found to shed recoverable, infectious virus. Interestingly, experimental infection did not reproduce the high mortality and severe clinical disease observed during the outbreak in the Philippines^4,20^. Likewise, results from our study support that asymptomatic infection with RESTV can occur in pigs and that these animals may pose a transmission risk to other susceptible animals or a risk to humans in close contact^16,20^.

Recently, a second series of experimental pig inoculation studies were performed by Haddock et al.^10^, also using a pig-derived RESTV isolate, investigating potential age-dependent susceptibility to infection and subsequent development of clinical disease. To accomplish this, three-, five-, and seven-week-old Yorkshire cross pigs were subjected to oronasal inoculation that led to clinical disease as early as three days post-inoculation (dpi) and progressed to severe respiratory distress by six dpi in all inoculated animals, regardless of age. Several animals in this study reached early humane endpoints and were subsequently euthanized, but those animals surviving the acute phase rapidly recovered by sixteen dpi. Systemic infection was evident by a detectable viremia in all animals and virus was found in oral, nasal, and rectal swabs at various levels and timepoints. High viral loads were detected in the lungs and associated lymph nodes, ranging from 10^4^ to 10^9^ TCID_50_ per gram of tissue, and low viral titers were also detected in the liver and spleen^10^. Haddock et al*.* proposed that infection outcome was not a factor of age, but could be affected by pig breed, pig genetics, or the effect of co-infection with unrelated respiratory or non-respiratory pathogens that could not be discerned^10,20^. It was noted in the Haddock et al*.* study that the inoculation dose used was ten-fold lower (10^5^ compared to 10^6^ TCID_50_) and that over-challenge of animals may have attenuated the development of clinical disease in the first study^10,20^.

In the study reported here, experimental inoculations were performed in domestic pigs using a wild-type, non-human primate-derived RESTV isolate using an oronasal dose of 10^6^ pfu total per pig; and we describe the associated susceptibility and pathogenesis associated with infection that resulted without the need for adaptation of the virus to the pig. The spectrum of clinical disease manifested was profiled, ranging from asymptomatic to severe respiratory distress, that both supports and contradicts previous reports. Additionally, we describe the first experimental transmission between infected pigs and a co-housed, naïve pig, as well as describing an assay for the detection of RESTV-specific IgA in both nasal wash fluid and non-invasive, group oral fluids. Based on this evidence, we hypothesize that it is possible that disease outcomes in domestic pigs experimentally inoculated with RESTV can be a result of the isolate used and that direct NHP-to-pig transmission may occur as a component of natural infection cycles.

## Results

### Evaluation of host susceptibility to infection

**Figure 1:**
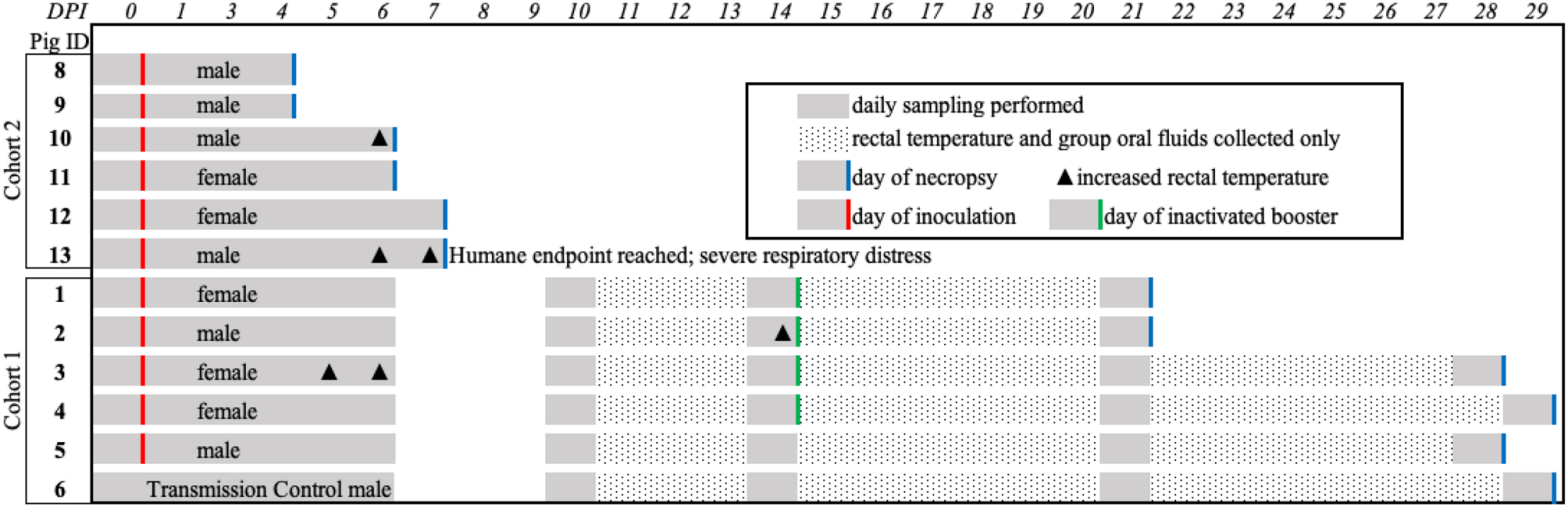
Study design, sample collection, and occurrence of increased rectal temperatures. Two cohorts of pigs were oronasally inoculated with a non-human primate-derived isolate of Reston virus (RESTV) while under general anesthesia at zero days-post-inoculation (dpi). Indicated animals were booster inoculated intramuscularly at fourteen dpi with 1 × 10^6^ pfu total UV-inactivated Reston virus. Daily sampling occurred zero to six, ten, fourteen, twenty-one, and twenty-eight or twenty-nine dpi for cohort one and zero to seven dpi for cohort two. Sampling included rectal, oral, and nasal swabs, nasal wash, and blood collection. A cursory physical exam, rectal thermometry, and aural pulse oximetry were performed on each animal while under anesthesia at indicated sampling time points. The "transmission control", Pig 6, was sham inoculated with media at zero and fourteen dpi and Pig 5 received a sham booster inoculation at fourteen dpi. Rectal body temperature was considered increased when greater than 40.0 °C. Animals were observed at least twice daily for development of clinical disease.

A series of studies were conducted to confirm susceptibility and to characterize the development of clinically apparent disease, viral shedding, host immune response, and tissue involvement in domestic pigs to a Reston virus isolate that had not been adapted to this host (Fig. 1). After oronasal inoculation, animals (n = 11) were group-housed by cohort in an open floor pen setting without barriers to peer interaction (Supplement Fig. 1). In the first cohort, a single naïve animal was sham inoculated and utilized as a “transmission control”. For viral inoculation, we generated a well-characterized challenge virus stock of RESTV isolated from the serum of a cynomolgus macaque involved in the 1989 outbreak in Reston, Virginia, USA (USA_VA_1989, 813168)^4,11^. The sequence obtained from whole genome sequencing of the inoculum stock was found to cluster with the parent sequence (GenBank accession KY798005)^11^.

Pigs inoculated with this NHP-derived isolate of RESTV (n = 11) demonstrated a spectrum of clinically discernable disease, ranging from asymptomatic as the most common to respiratory distress as the most severe. Four animals exhibited a febrile state, as defined for this study as ≥ 40.0 °C, lasting for twenty-four to forty-eight hours with three of the four animals’ temperatures peaking at six dpi (40.4–41.6 °C) and the fourth animal having only a slightly increased temperature (40.1 °C) at fourteen dpi (Fig. 1 and Supplement Fig. 2a). An increased rectal temperature was not detected in the transmission control animal at any point during the study. There were no significant changes noted in pulse oximetry readings observed during sedation (Supplement Fig. 2b,c) or in the evaluated clinical laboratory parameters (Supplement Fig. 3a–c). At seven dpi, one animal (Pig 13) reached a humane endpoint and was humanely euthanized (Fig. 1). This pig was febrile (41.6 °C), physically depressed, reluctant to move, and exhibited severe respiratory distress, including sternal positioning, dyspnea, and tachypnea (Supplement Fig. 2d). Cyanosis was not apparent. Regurgitated food was also present in the room and on this pig’s snout.

### RESTV infected pigs shed infectious virus predominately by the nasal and oral routes

**Figure 2:**
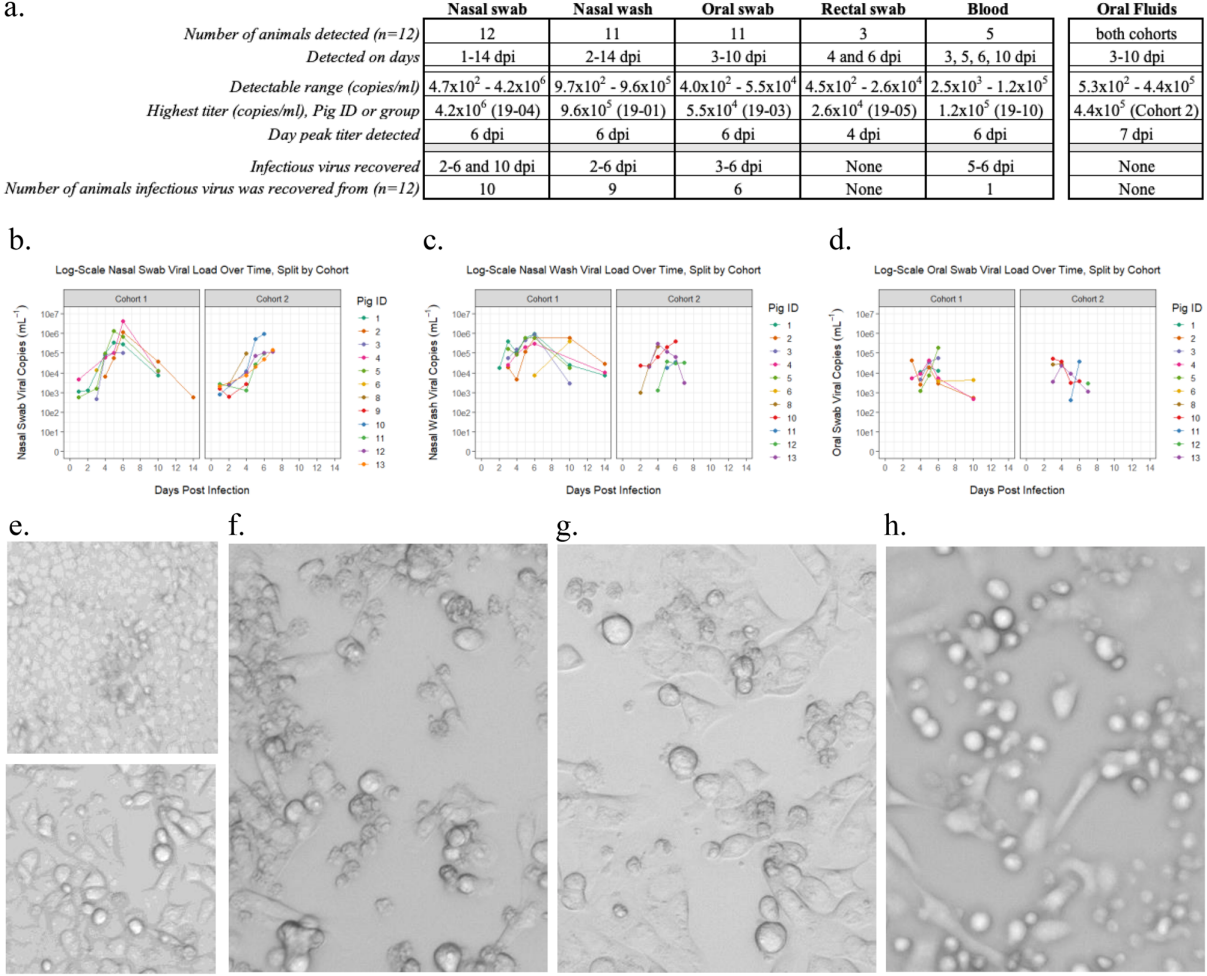
Reston virus-infected pigs shed infectious virus predominately by the nasal and oral routes. Reston virus (RESTV) was detected by rRT-PCR in nasal wash, blood and nasal, oral, and rectal swabs, as well as group oral fluid collected by rope chew, from pigs in both cohorts. Infectious virus was recovered from all sample types, except for rectal swabs and oral fluids. (**a**) Summary of all findings by sample type. Data from nasal swab (**b**), nasal wash (**c**), and oral swab (**d**) sample viral load, as detected by rRT-PCR (gene copies/mL), are plotted by individual animal and day post infection. The presence of infectious virus in each sample was evaluated by virus isolation utilizing cell culture with evaluation for the presence of cytopathic effect (CPE). Light microscopy images representative of these findings are shown: (**e**) negative (top panel) and positive (bottom panel) controls; positive CPE for nasal swab (**f**), nasal wash (**g**), and oral swab (**h**) samples.

Shedding of RESTV in the study pigs was predominately via the oral and nasal routes as detected by rRT-PCR and isolation of infectious virus in cell culture (Fig. 2a). Viral RNA was detectable from one to fourteen dpi in nasal swabs and nasal washes with a range of 4.7 × 10^2^ to 4.2 × 10^6^ copies/ml and 9.7 × 10^2^ to 9.6 × 10^5^ copies/ml, respectively (Fig. 2b,c and Supplement Fig. 4a,b). Live virus was recovered from nasal swabs at least once from ten of twelve animals from two to ten dpi (Fig. 2f and Supplementary Fig. 4a) and in the nasal wash fluid from nine of twelve animals between two and six dpi (Fig. 2g and Supplement Fig. 4b). The peak titers for both nasal swabs and wash occurred at six dpi. Viral RNA was detectable in oral swabs from three to ten dpi with peak levels (5.5 × 10^4^ copies/ml) at three dpi (Fig. 2d). Infectious virus was recovered from oral swab material from three to six dpi for six of twelve animals (Fig. 2e,h and Supplement Fig. 4c). RESTV RNA was identified in group oral fluids from four to ten dpi for cohort one and three to seven dpi for cohort two. A peak titer of 4.4 × 10^5^ copies/ml was detected at seven dpi from group oral fluids (Fig. 2a), but we were unable to recover live virus from any time point. Not unexpectedly, the group oral fluid samples were heavily contaminated with bacteria and fungus contributing to the lack of virus recovery from these samples (Supplement Fig. 4f). Shedding of virus in feces or detection in blood appeared to be limited. RESTV RNA was detected only once in rectal swabs from each of three animals, two at four dpi and one at six dpi, and infectious virus was not recoverable from these samples (Supplement Fig. 4d). RNA from RESTV was detected sporadically in blood from five of twelve animals between three and ten dpi, with live virus recovered only twice at five and six dpi, from a single animal (Supplement Fig. 4e). The transmission control animal was positive for RESTV RNA with recovery of live virus from nasal swab at three dpi and by both oral swabs and nasal washes at six and ten dpi (Supplement Fig. 4a–e).

### Infected pigs develop a robust RESTV-specific antibody response as indicated by IgA, IgM, and IgG

**Figure 3:**
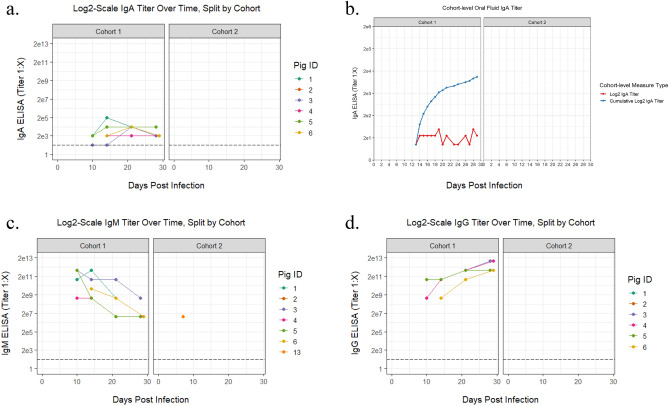
Pigs experimentally infected with Reston virus develop a robust virus-specific antibody response. Serial serum, nasal wash, and oral fluid samples were collected from Reston virus (RESTV)-infected pigs to develop a timeline concerning the development of virus-specific antibodies as measured by ELISA per the study design described in Fig. 1. Titer data is log-transformed back to base scale, split by cohort, and provided for each individual animal (**a**, **c**, **d**), or for each group (**b**). Individual animal IgA titers for nasal wash fluid samples were detectable at ten days post infection (dpi) with all animals having a detectable titer by fourteen dpi (a). IgA titers were also detectable in group oral fluid (OF) collected by rope chew utilizing the same ELISA (**b**). The titers for OF are log-transformed, then summed for each day ("sum") and also shown as "cumulative" values (summed from zero dpi, retaining the value from the previous days). The cumulative titer (blue line) shows that the rate of IgA antibody detection was greatest from fourteen to twenty dpi and then remained relatively constant after that point. IgA antibodies were not detected in the OF collected from cohort two. RESTV-specific IgM (**c**) and IgG (**d**) antibodies in sera were measured with all inoculated animals in cohort one having detectable titers at ten dpi and a single animal in cohort two having detectable IgM at seven dpi.

RESTV-specific IgA was detectable between ten and twenty-nine dpi in both individual nasal wash fluid and the group oral fluids collected by rope chew and virus-specific IgA was detected utilizing an ELISA developed with commercially available components. IgA was detected in the nasal wash fluid of three of five inoculated animals at ten dpi and five of five inoculated animals by fourteen dpi (Fig. 3a). Once detected, animals remained positive until the time of euthanasia. IgA was detected in the sham-inoculated transmission control pig (Pig 6) by fourteen dpi, and this animal also remained positive until the end of the study at twenty-nine dpi (Fig. 3a). Group oral fluid samples were positive for IgA beginning at thirteen dpi and remained positive until twenty-nine dpi, less days twenty-two and twenty-five when IgA was not detected (Fig. 3b). The detectable levels in the group samples were variable, ranging in titers from 1:4 to 1:6 (Fig. 3b). RESTV-specific IgM and IgG were measured in sera using a previously described protocol^10^. IgM was detected in a single animal in cohort two at seven dpi, and all five inoculated animals in cohort one were positive at ten dpi for IgM and IgG. All animals remained positive until euthanasia, albeit with decreases in IgM titer by the end of the study (Fig. 3c,d). The transmission control animal became positive for both serum IgM and IgG by fourteen dpi and remained positive until euthanasia. Interestingly, virus-specific neutralization activity was not detected in any animal at any time point when screened by a modified plaque reduction neutralization test.

### Gross pathology, histopathology, and recovery of infectious virus from tissues

**Figure 4:**
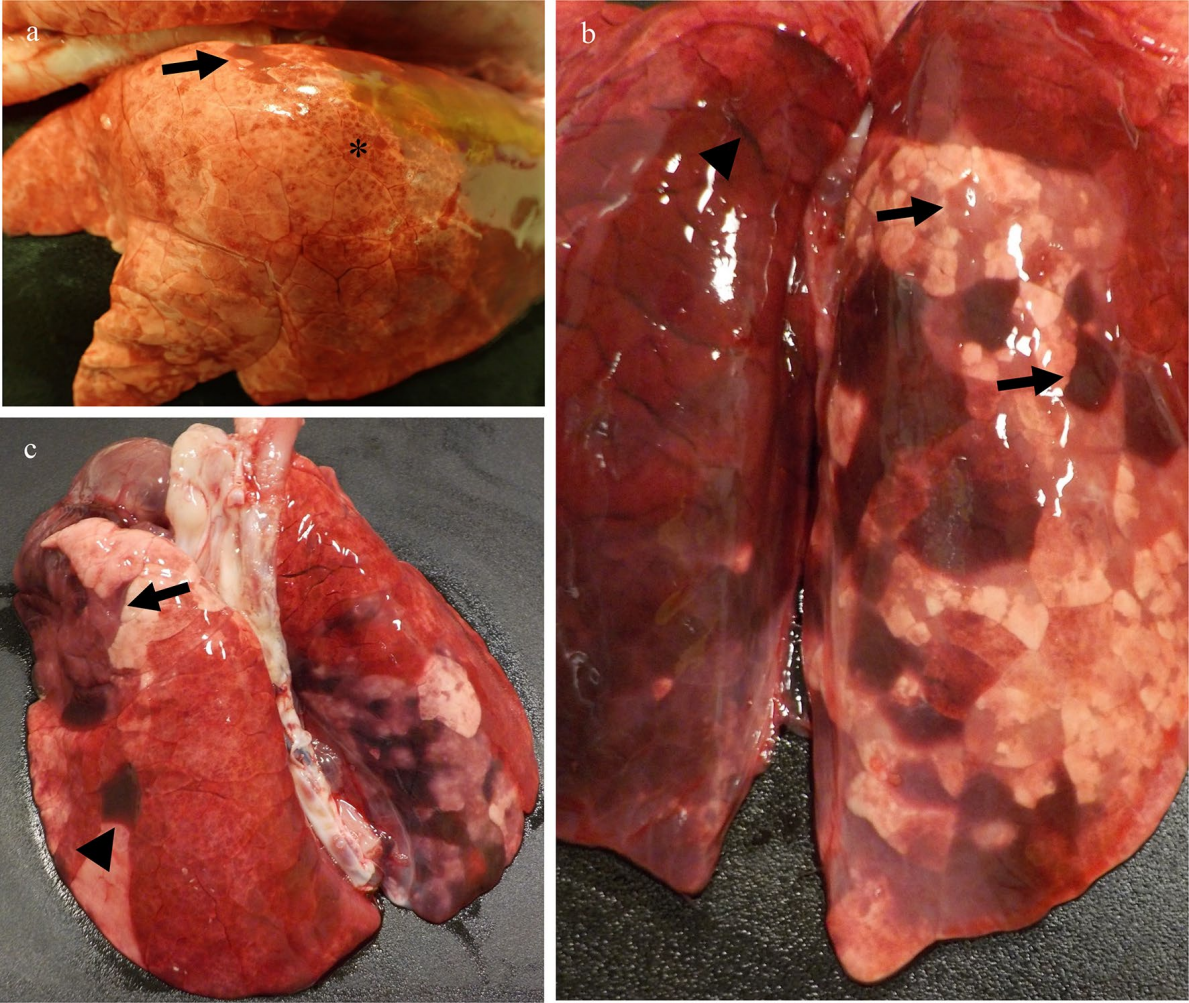
Gross pathology of the respiratory system in Reston virus-infected pigs. Scattered firm, red areas of consolidation consistent with pneumonia (arrow) and patchy congestion (*) were grossly evident in the lungs of pigs euthanized at four days post-infection (dpi) (**a**). The lung pathology progressed by six dpi, one animal displayed lesions that included widened interlobular septa interpreted as pulmonary edema (arrowhead) with numerous pink to red areas of pneumonia occurring in a lobular pattern (arrows) (**b**). At seven dpi, one pig was humanely euthanized due to severe respiratory distress, including tachypnea and dyspnea. During necropsy, this animal’s lungs were wet and heavy with firm, depressed and red areas of pneumonia in both cranial (arrow) and caudal lung lobes (arrowhead) (**c**). Samples of the lung tissue sank when placed in formalin.

Animals were euthanized at pre-determined time points or upon reaching an IACUC-approved humane endpoint. Gross lesions noted during necropsy were predominately observed in the lungs and were consistent with the development of interstitial pneumonia during the acute phase of the disease. At four dpi, there were extensive areas of congestion and scattered firm red lobules in the pulmonary tissue (Fig. 4a). By six and seven dpi there was evidence of pulmonary edema (Supplement Fig. 2e) and multifocal to coalescing firm red areas of consolidation occurring in a lobular pattern indicating the presence of pneumonia (Fig. 4b,c). Significant gross lung lesions were not noted at later time points. A mild enlargement of various lymph nodes could be observed in some of the animals throughout the study, including the mandibular and tracheobronchial lymph nodes.

**Figure 5:**
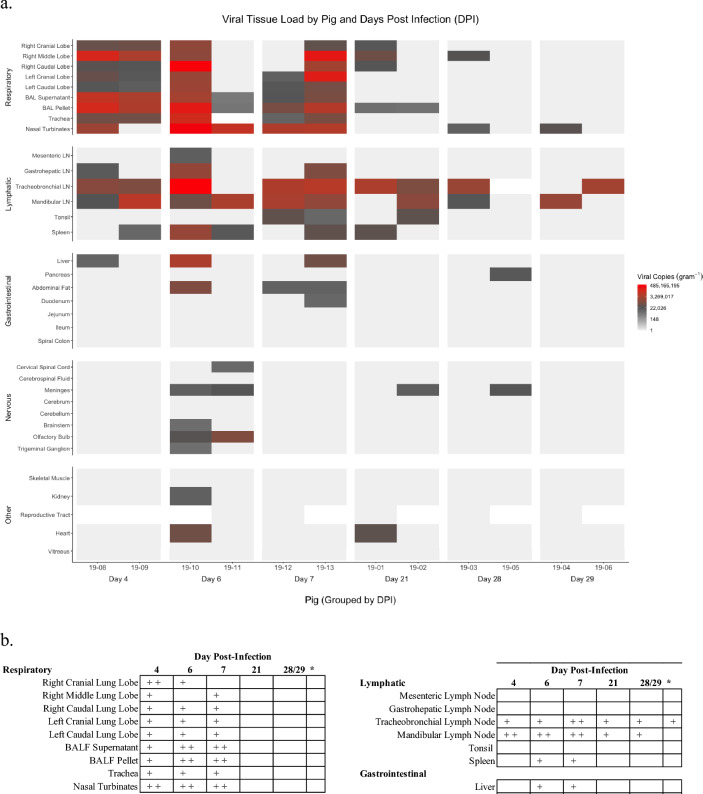
Distribution, quantification, and replication of Reston virus in tissues of experimentally infected pigs. The heatmap (**a**) depicts the mean of technical replicates of Reston virus (RESTV) copies per gram of tissue as determined by rRT-PCR for experimentally infected pigs by day post infection. Tissues are grouped by general anatomic system, showing that the virus predominately effects respiratory and lymphatic tissues. The recovery of infectious virus by cell culture isolation of tissue samples is summarized (**b**). Successful recovery of virus from an animal is indicated by a positive sign (+) for each animal necropsied at the listed timepoint. dpi, day post-inoculation; BALF, bronchoalveolar lavage fluid.

The highest viral RNA yields, determined by rRT-PCR, were from the respiratory and lymphatic tissues including all lung lobes, trachea, nasal turbinates, tonsil, and lymph nodes from numerous locations, as well as from bronchoalveolar lavage fluid (BALF) (Fig. 5a). Viral RNA was detected in the spleen, liver, meninges, olfactory bulb, and hearts of two animals. Additionally, viral RNA was detected in the pancreas, duodenum, cervical spinal cord, brainstem, trigeminal ganglion, and kidney in one animal each. Viral RNA was also detected in the abdominal fat of three animals sampled at six and seven dpi, though infectious virus was not recoverable nor was antigen detected by IHC in these samples. Tissue yields of infectious virus were inconsistent, and successful recovery of infectious virus was limited to respiratory and lymphatic tissues, as well as the isolation of virus from the liver of two animals (Fig. 5b).

**Figure 6:**
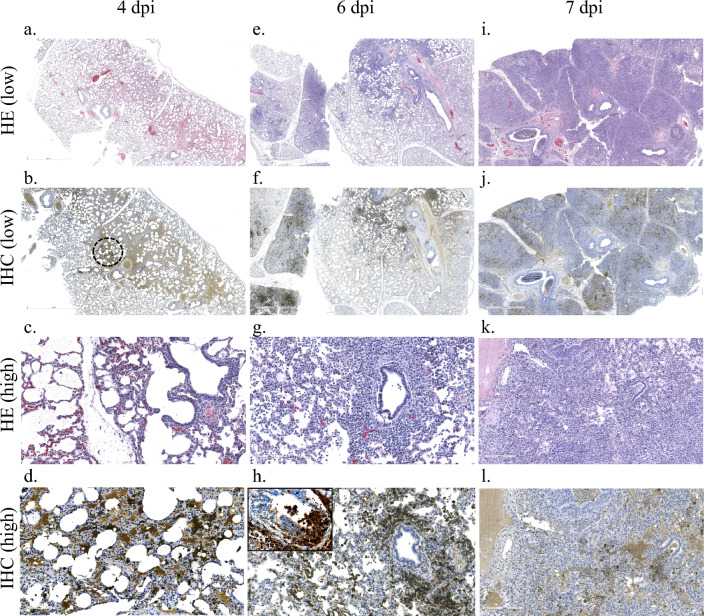
Histopathology and immunohistochemistry findings in the lungs of Reston virus-infected pigs. Mild interstitial inflammatory infiltrate, as well as mild alveolar and interlobular edema (**a**, **c**) in the pulmonary interstitium was observed in the lungs of both pigs necropsied at four days post inoculation (dpi). Abundant viral antigen was detected by immunohistochemistry (IHC) and was most often associated with interstitial and alveolar macrophages, as well as being present free in the capillaries and alveolar spaces (**b**, **d**). Panel b shows an example of non-specific IHC reaction with edema fluid that was noted at several time points (light staining outside of the circled area). In one animal necropsied at six dpi, there were multifocal areas of moderate interstitial pneumonia characterized by the expansion of alveolar septa with inflammatory cells, primarily macrophages, as well as peri-bronchiolitis and an increase in alveolar macrophages (**e**, **g**). Abundant viral antigen was detected by IHC and visually correlated with the presence of lesions (**f**). The distribution of antigen was similar to that observed at four dpi with the additional association with peri-bronchiolar inflammatory cells (**h**) and bronchiolar epithelium (**h**, insert). An animal with severe respiratory distress was euthanized and necropsied at seven dpi. In this animal, there was severe, diffuse interstitial pneumonia (**i**) with neutrophilic exudate in the alveolar spaces and bronchiolar lumens, as well as alveolar edema and scattered areas of necrosis (**k**). There was extensive distribution of viral antigen throughout the section (**j**) primarily found within macrophages and associated with inflammatory exudate (**l**). Tissues originated from Pig 8 (**a**–**d**), Pig 10 (**e**–**h**), and Pig 13 (**i**–**l**).

**Figure 7:**
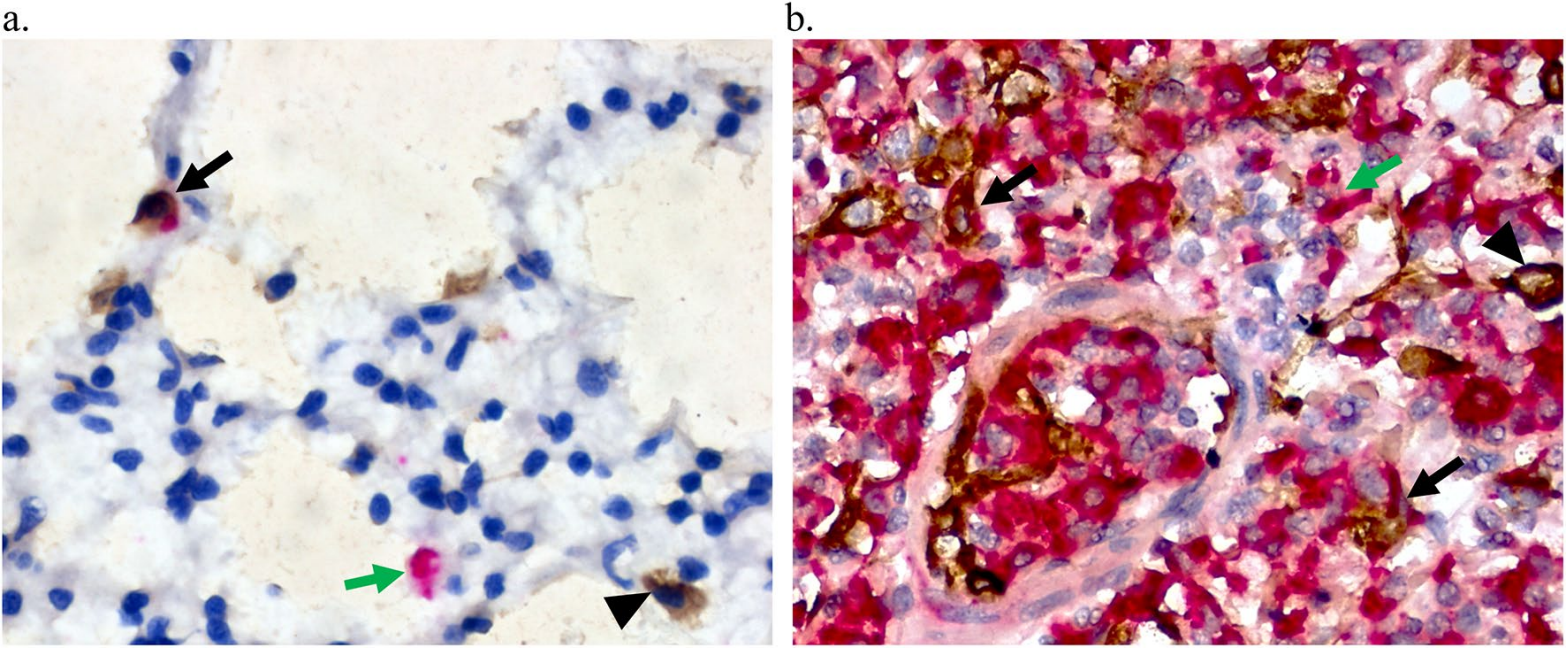
Pulmonary macrophages and pneumocytes are infected during Reston virus infection in domestic pigs. A combination of immunohistochemistry (IHC) and in situ hybridization (ISH) techniques were used to identify Reston virus (RESTV) infection of cell types in the lung tissue of experimentally infected pigs. Macrophages (stained brown; **a**, arrowhead) were identified by IHC and infected cells (stained pink; **a**, green arrow) were identified by ISH. Infected macrophages were observed primarily within the alveolar septa (stained brown and pink; **a**, black arrow). Epithelial cells (stained brown; **b**, arrowhead) were identified by IHC and infected cells (stained pink; **b**, green arrow) were identified by ISH. Numerous infected pneumocytes (stained brown and pink; **b**, black arrow) were identified throughout the lung section. Tissues originated from Pig 10.

Mirroring the results of viral RNA detection and infectious virus isolation, as well as the gross pathology noted at necropsy, histopathology and immunohistochemistry (IHC) findings were most prominent in the lungs and the associated draining lymph nodes. Pulmonary manifestations of RESTV infection appeared to progress from moderate to severe interstitial pneumonia by seven dpi (Fig. 6). The histopathological changes observed included interstitial mononuclear cellular infiltrate with alveolar macrophages, peri-bronchiolitis, interlobular and alveolar edema, and accumulation of neutrophils in alveolar spaces and bronchiolar lumens (Fig. 6a,c). By four dpi, viral antigen was distributed within interstitial and alveolar macrophages, as well as free antigen in the capillaries and alveolar spaces (Fig. 6b,d). By six dpi, viral antigen was present in bronchiolar inflammatory cells and in the bronchiolar epithelium (Fig. 6e–h). At seven dpi, the distribution of antigen was extensive, and it was found primarily in macrophages and in inflammatory exudate (Fig. 6i–l). A combination of IHC and ISH staining was utilized to confirm the presence of RESTV genomic material within both pulmonary macrophages and pneumocytes. Infected macrophages were readily apparent in the alveolar septa (Fig. 7a) and infected pneumocytes were found throughout the evaluated lung tissue sections (Fig. 7b).

**Figure 8:**
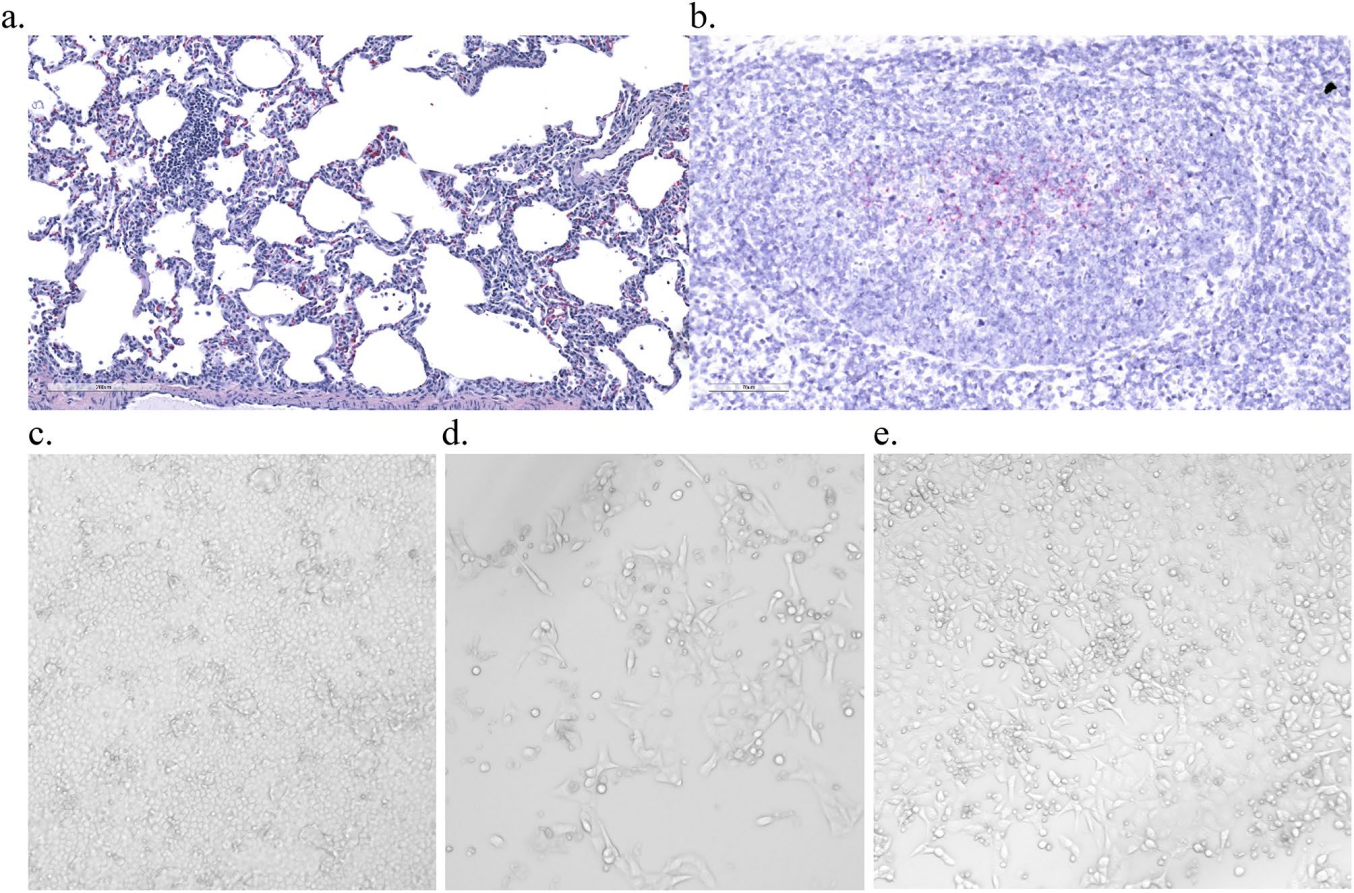
Histopathology and tissue in situ hybridization (viral genomic RNA) findings in Reston virus-infected pigs at twenty-one and twenty-nine days post-inoculation. The lungs of one of two animals euthanized and necropsied at twenty-one days post-infection (dpi) showed multifocal areas of mild interstitial pneumonia and an increased presence of alveolar macrophages on histopathologic examination (**a**). No other significant lesions were noted at this time point and neither viral antigen nor viral RNA could be detected in this tissue by either immunohistochemistry (IHC) or in situ hybridization (ISH). No significant histopathologic lesions were noted at twenty-nine dpi, however viral RNA was detected by ISH in the tracheobronchial lymph node of the sham-inoculated, transmission control animal (**b**). Viral antigen could not be detected in this tissue by IHC. Infectious virus was recovered from this sample by cell culture isolation as determined by the development of cytopathic effect (CPE) in Vero cell monolayers. Representative images of negative control (**c**), positive control (**d**), and sample (**e**) showing CPE. Tissues originated from Pig 2 (**a**) and Pig 6 (**b**).

Lesions were observed in the lymph nodes, nasal turbinates, and trachea consisting of mild to moderate submucosal inflammation that corresponded with areas of viral antigen distribution detected by IHC (Supplement Figs. 6, 7 and 8). Both pigs necropsied at twenty-one dpi had no abnormalities noted on gross exam, but one animal showed evidence of mild interstitial pneumonia with an increased presence of alveolar macrophages on histopathologic evaluation (Fig. 8a). Neither viral antigen nor genomic material were detected by either IHC or in situ hybridization (ISH). Interestingly, RESTV genomic RNA was detected in the tracheobronchial lymph node of the transmission control animal (Pig 6) necropsied at day twenty-nine (Fig. 8b). Infectious virus was recovered from this sample as determined by the development of cytopathic effect (CPE) in Vero cell monolayers (Fig. 8c–e).

### Experimentally infected pigs can transmit virus to naïve pigs

In cohort one, we utilized a sham-inoculated “transmission control” pig as a proof-of-concept for virus transmission from experimentally inoculated pigs to a co-housed, naïve animal. This animal did not demonstrate clinical disease, but transmission and subsequent infection of this animal was shown by detection of virus in both clinical samples and tissues. Nasal swab samples were positive by rRT-PCR only at day three, but infectious virus was recovered from this sample (Supplementary Fig. 4a). Nasal wash and oral swab samples were positive by rRT-PCR and for recovery of infectious virus at day six and at day ten (Supplement Fig. 4b,c); unfortunately, animals in this cohort were not sampled at seven to nine dpi. Seroconversion demonstrated by detection of IgM and IgG in sera and IgA in nasal wash fluid occurred by day fourteen in this animal (Supplementary Figs. 5a,c,d). There was limited detection of virus in tissues with only the tracheobronchial lymph node having viral RNA detectable by rRT-PCR (Fig. 5a); this sample was also positive by ISH and infectious virus was recoverable by cell culture isolation (Fig. 7b,e).

### Susceptibility of pigs did not require species-specific adaptation of RESTV

Interrogation of virus sequences in tissues revealed limited changes compared to the challenge virus stock indicating that pig-adaptation of the virus was not required for infection or associated with development of disease. Sequencing revealed the challenge virus stock to have a 99.98% genome-wide nucleotide identity with the original isolate, USA_VA_1989 (GenBank accession KY798005). Genome sequences recovered from terminal tissue samples were identical to the that of the inoculum (Supplement Fig. 9). When the genomes of the inoculum and virus isolated from terminal tissue samples were aligned against the annotated parent genome, USA_VA_1989, a total of seven single nucleotide polymorphisms (SNPs) were detected based on the available annotation of the reference isolate (KY798005). Two SNPs were discovered in the coding region for nucleoprotein (NP) and two in viral protein 40 (VP40), with the remaining three found in non-coding regions (Supplement Fig. 9). When comparing the genomes from the terminal tissue samples to the inoculum, one animal’s tracheobronchial lymph node (Pig 6) had a novel SNP resulting in a non-coding variant. Mandibular lymph node from Pig 8 yielded a genome that had two novel SNPs both located in coding regions, one in the region coding the NP and one located in the VP40 coding region. No other variants were noted in any other tissue sample when compared to the genome of the inoculum (Supplement Fig. 9).

## Discussion

Non-human primates and pigs are the only two host animal species, other than humans, that have been shown to be naturally infected by RESTV^4,21^. Both are thought capable of serving as spillover hosts from bats, the presumed reservoir host, followed by sustained, intra-species transmission^4,11,13,21^. Our study found that domestic swine are susceptible to NHP-derived isolates of RESTV without the need for host species-specific adaptation of the virus. Of the eleven pigs inoculated, all had virus detected by rRT-PCR being shed in oronasal secretions indicating that all animals were susceptible to infection. Ten of the eleven had infectious virus recovered from either nasal swab, nasal wash, and/or oral swab between two and ten dpi. Interestingly, clinical disease presented as a spectrum of manifestations, with only three animals exhibiting a transient increase in rectal temperature during the acute phase of infection between five to seven dpi, and only a single animal having exhibited appreciable clinical signs, while all other pigs remained clinically normal. All inoculated animals that survived to ten dpi had developed detectable levels of both IgM and IgG antibodies specific to RESTV, which remained detectable until the end of the study at twenty-nine dpi. RESTV-specific IgA antibodies were also detectable by ELISA in nasal wash fluid between ten and fourteen dpi.

The utility of group oral fluids as a diagnostic tool for RESTV was also evaluated. Use of this sample matrix has been expanding, and the non-invasive approach it allows is readily supported by the pig production industry^22^. RESTV RNA was readily detectable in oral fluids from both study groups between three and ten dpi, but attempts at recovery of infectious virus from this sample type was not successful. Difficulties in the recovery of infectious virus from the oral fluid matrix is not unusual and will most likely require further optimization^22^. Of note, virus-specific IgA antibody was readily detectable in group oral fluids between thirteen and twenty-nine dpi using the ELISA described for nasal wash fluid. Though further validation would be required, these findings support the use of this sampling method and should be considered in future surveillance and response monitoring efforts.

The clinical results of this study both support and contradict previous reports of experimental infection of domestic pigs with RESTV. One study reported only asymptomatic disease and the other reported all pigs developing severe respiratory distress, with both studies reporting similar pathology, antibody response, and shedding of infectious virus. Both studies utilized pig-derived RESTV isolates originating from the Philippine 2008 epizootic^10,20^. The most recent pig study with all pigs developing severe respiratory distress characterized their inoculum as isolate PHL_A_2008 (GenBank: KY798011 and MT796851)^10^. This virus was isolated from a lymph node collected from a pig on a production farm in Pandi, Bulacan, on the island of Luzon on June 4, 2008, and was submitted as a diagnostic sample to the United States Department of Agriculture’s Foreign Animal Disease Diagnostic Laboratory (FADDL). Infectious virus was subsequently isolated after a single passage in Vero E6 cells^4,11^. Three distinct RESTV isolates were recovered from this outbreak, each displaying a high degree of phylogenetic diversity suggesting that each virus originated from an independent spillover event from the unknown reservoir host leading to sustained pig-to-pig transmission^4,11^. As the sequence of the virus used to inoculate animals in the original pig susceptibility study is not available (personal communication with G. Marsh, 2021), it is not possible to know which of the original isolates from the epizootic or from which farm this represented^20^. Since these viruses have previously been shown to be the result of independent spillover events^4,11^, we theorize that there may be variations in the individual isolate’s ability to cause disease in pigs. Supporting this, the RESTV isolate used in the current study is derived from an NHP imported from the Philippines to Reston, Virginia, during the 1989 outbreak (GenBank Accession KY798005)^11^. Specifically, this virus was isolated after two passages in Vero E6 cells from the serum of NHP #53, a cynomolgus macaque, from Room F of the Hazelton Primate Quarantine Facility^11^.

To evaluate intra-species transmission of the NHP-derived RESTV isolate, a naïve, sham-inoculated pig was co-housed with the infected cohort in Study 1. Though this pig did not demonstrate clinical disease, transmission to and infection of this animal was shown by detection of virus in clinical samples, including nasal swabs, nasal washes, and oral swabs. This animal was also shown to seroconvert by detection of IgM and IgG in sera and IgA in nasal wash fluid. Though virus genome was detected by both rRT-PCR and ISH and infectious virus was isolated in only one tissue from this animal, we hypothesize that this limited tissue detection may be secondary to sample timing as this animal was necropsied at only twenty-nine days after the co-housed animals were inoculated. This is the first report of experimental transmission of RESTV between pigs, and we feel that these findings serve as a proof-of-concept justifying the need for further transmission studies in the future.

Altogether, these findings indicate that domestic pigs are susceptible to infection with a wild-type, NHP-derived isolate of RESTV leading to shedding of infectious virus into the environment with a spectrum of clinical disease ranging from no clinical signs to severe respiratory distress. Therefore, it is reasonable to hypothesize that natural infection of domestic pigs does not require species-adaptation and could arise directly from exposure to an infected NHP. Further supporting this conclusion, deep sequencing of the RESTV genome revealed no mutations in virus isolated from terminal tissue samples when compared to the challenge virus stock used for inoculation, indicating that these results are likely not secondary to virus mutation or host adaptation. We propose that the variation in experimental infection outcomes of the two previously reported studies and the study reported here could be associated with the host species origin of the virus, species-adaptation status, or cell culture passage history of the virus isolate itself.

## Methods

### Ethics and animal welfare statement

All infectious work with RESTV, including sample inactivation, was performed in the Containment Level 4 laboratory (CL4) in accordance with the policies and protocols outlined by the Canadian Science Centre for Human and Animal Health Institutional Biosafety Committee. All animal work was performed in strict accordance with the Canadian Council for Animal Care under the approval and oversight of the facility Institutional Animal Care and Use Committee (IACUC; Animal Use Document #19-006). As pigs are social animals, they were group housed in an open pen setting throughout the study, under controlled conditions of humidity, temperature, and light (12-h light/12-h dark cycles). Food and water were available ad libitum. Animals were fed a commercial pig diet, as well as fruits and vegetables, and also provided environmental enrichment with daily monitoring by trained personnel. Humane endpoints were approved by the IACUC and specified when animals should be humanely euthanized.

### Study design

A total of fourteen domestic, American Yorkshire crossbred pigs (*Sus scrufa domesticus*) (eight castrated males and six females, age six weeks), sourced from a high-health, commercial herd, were utilized with seven pigs being used in each of two studies conducted (Fig. 1). Following one week acclimatization, one pig was sampled and necropsied prior to infection in each study to serve as a farm control and source of negative samples and tissues. The remaining six pigs were each challenged oronasally with 1.0 × 10^6^ pfu total of RESTV in 3.0 mL media distributed as 1.0 mL in each nostril and 1.0 mL placed in the distal pharynx utilizing a sterile, tomcat-style catheter.

The first study (n = 6) lasted twenty-nine days to allow for the monitoring of the antibody development, as well as for the production of high titer sera for use by the diagnostic laboratory. For this reason, a "prime-boost" strategy was undertaken; and four of the six animals were booster inoculated intramuscularly at fourteen dpi with 1.0 × 10^6^ pfu total of a UV-inactivated, whole virus preparation produced from the inoculum virus stock in 0.5 mL media. The challenge dose and inactivation were confirmed by back-titration as described below. Necropsies were performed at twenty-one, twenty-eight, and twenty-nine dpi with two pigs at each time point. One animal, Pig 6, was co-housed, sham-inoculated, and sham-boosted as a proof-of-concept for intra-species transmission of the virus ("transmission control"). The second study (n = 6) was designed to evaluate the acute phase of infection with necropsies of two animals each performed at four, six, and seven dpi. The final two pigs were originally planned to be necropsied at eight dpi, but one animal developed severe respiratory distress, a pre-designated humane endpoint, resulting in the euthanasia and necropsy of both animals a day earlier.

After inoculation, animals were monitored twice daily for development of clinical signs. Extensive individual sampling was undertaken at time points outlined in Fig. 1. Each sampling involved anesthetizing the animal, conducting a cursory physical exam, aural pulse oximetry, and the collection of nasal wash fluid, as well as oral, nasal, and rectal swabs. Blood was collected for clinical pathology evaluation, including complete blood cell counts, serum chemistries, and blood gas analysis, as well as for the detection of viremia and the development of antibodies. Group rope chews were used daily to collect oral fluid samples from the study group and as a source of enrichment for the animals (Supplement Fig. 1).

Animal numbers were not based on power analysis but on the limitations of the CL4 animal room size. Group assignment (day of euthanasia and necropsy) was based on randomization at the time of permanent animal identification assignment (ear tag). Pigs were confirmed negative for *Mycoplasma spp*., *Betaarterivirus suid 1*, and *porcine circovirus-1* and *-2* by PCR of 0 dpi and terminal samples following standardized procedures provided by the Manitoba Provincial Veterinary Diagnostic Laboratory.

This study is reported in accordance with ARRIVE guidelines under the constraints of CL4.

### RESTV stock and inoculum preparation

Stock virus was produced and titrated by an immunocytochemistry assay (described below) on confirmed bacterial and *Mycoplasma spp.*-free Vero 76, Clone E6 cells (ATCC, CRL-1586, Lot 58027482) in Dulbecco’s modified Eagle’s medium (DMEM) supplemented with 2% fetal bovine serum (FBS) and stored at  − 150 °C until utilized. Viral stocks were diluted to challenge dose in DMEM. The identity and purity of the RESTV isolate used in this study was verified by a next-generation sequencing (NGS) as a 1989 isolate originating from the serum of non-human primate #53 after passage in Vero cells^11^. The inoculum used in this study represents the fourth passage in the same cell line and was confirmed to have a 99.99% genome-wide nucleotide identity with *Orthoebolavirus restonense* isolate USA_VA_1989 (811952).

### UV-inactivation of booster inoculum

UV-inactivation of booster inoculum was performed in twelve-well plates exposed under a UV lamp (Analytica Jena US; UVS-28 EL Series UV Lamp) without the lid for one hr. After UV exposure, the booster inoculum was kept at 4 °C until use. Successful inactivation was confirmed by attempts at viral titration with immunocytochemistry performed as described below after each of two serial passages in Vero E6 cells demonstrated no cytopathic effects and no virus-specific staining.

### Viral titration determined by immunocytochemistry

Virus was titrated using a previously described immunocytochemistry assay on Vero E6 cells^23^. Samples were serially diluted (1:10) and incubated on confluent monolayers of Vero E6 cells in 96-well plates at 37 °C and 5% CO_2_ for one hour. 1.75% carboxymethylcellulose (CMC; Sigma) containing DMEM, 7.5% bovine serum albumin fraction V, 7.5% sodium bicarbonate, HEPES 1M, 0.4g/L 100 × folic acid, 200 mM L-glutamine, 11.0 mg/mL sodium pyruvate, and 100 × penicillin/streptomycin was added to each well. The plates were incubated for four days at 37 °C and 5% CO_2_ and then fixed for 24 h with 10% buffered formalin. The CMC overlay and formalin were removed and 0.3% Triton X-100 was used to permeabilize the cell monolayer after washing. An in-house generated, primary anti-RESTV nucleoprotein antibody was added at a pre-determined dilution of 1:2000, and the plates were incubated at room temperature with agitation. After incubation, the plates were washed with TBS-T (0.05% Tween-20) and deionized water; and an HRP-labeled, anti-mouse secondary antibody was applied (Dako, K4001). Following room temperature incubation and washing with TBS-T and water, substrate was added (Dako EnVision + Substrate System; K3468). The plates were washed with deionized water and allowed to dry before the stained plaques were counted with the aid of a stereo dissecting microscope.

### Daily sampling of animals

Oral, rectal, and nasal swabs were taken from each pig at the time points outlined in Fig. 1 under general anesthesia using Isoflurane and placed into sterile D-PBS containing the following antibiotics: streptomycin, vancomycin, nystatin, and gentamycin. Fluid was collected from a bilateral nasal wash with sterile D-PBS, and one of each of the following tubes was collected via jugular venipuncture: serum, sodium citrate, sodium heparin, and K3 EDTA (Fig. 1). While under anesthesia, a physical exam including rectal temperature and pulse oximetry (Massimo Rad 5) was conducted on each pig. Fluid from group rope chews was collected daily using a commercially available collection kit (Tego Swine Oral Fluids Kit, A100930, ITL BioMedical Animal Healthcare).

### Complete blood counts, blood chemistry, and blood gas analyses

Hematology was performed on a VetScan HM5 hematology analyzer (Zoetis, USA) using K3 EDTA-treated whole blood and the following parameters were evaluated: red blood cells, hemoglobin, hematocrit, mean corpuscular volume, mean corpuscular hemoglobin, mean corpuscular hemoglobin concentration, red cell distribution weight, platelets, mean platelet volume, white blood cells, neutrophil count (absolute (abs) and %), lymphocyte count (abs and %), monocyte count (abs and %), eosinophil count (abs and %), and basophil count (abs and %). Blood chemistries were evaluated on a VetScan VS2 (Zoetis, USA) with the Preventative Care Profile Plus rotor (Zoetis) using sodium heparin treated whole blood; and the following parameters were evaluated: glucose, blood urea nitrogen, creatinine, calcium, albumin, total protein, alanine aminotransferase, aspartate aminotransferase, alkaline phosphatase, total carbon dioxide, potassium, sodium, chloride, globulin, and total bilirubin. Sodium heparin treated blood was also used to analyze blood gases, which were performed on an iSTAT Alinity V machine (Zoetis, USA) using a CG4 + cartridge (Zoetis, USA) to measure the following parameters: lactate, pH, total carbon dioxide, partial pressure carbon dioxide, partial pressure oxygen, soluble oxygen, bicarbonate, and base excess. All analyses were performed according to the manufacturer’s instructions.

### Necropsy and post-mortem sampling

Necropsies were performed after euthanasia via pentobarbital overdose, confirmation of death, and exsanguination by femoral artery laceration. The following tissues were collected and split between 10% neutral-buffered formalin and fresh tissue: skin, skeletal muscle, abdominal fat, liver, spleen, pancreas, duodenum, jejunum, ileum, spiral colon, kidney, gastrohepatic and mesenteric lymph nodes, right cranial lung lobe, right middle lung lobe, right caudal lung lobe, left cranial lung lobe, left caudal lung lobe, trachea, heart, tracheobronchial lymph nodes, cervical spinal cord, meninges, cerebrum, cerebellum, brainstem, olfactory bulb, nasal turbinates, submandibular lymph nodes, tonsil, trigeminal ganglion, and the entire eye. The reproductive tract (uterus and ovaries) were collected *en bloc* in female animals. Cerebrospinal fluid, urine, vitreous fluid were also collected.

### Histopathology

Histopathology, immunohistochemistry, and in situ hybridization were performed based on positive results during RESTV RNA detection. After fixation and inactivation of tissues in 10% neutral phosphate buffered formalin for a minimum of seven days, tissues were trimmed and removed from the maximum containment laboratory, routinely processed, and stained with Gill’s hematoxylin and eosin (HE) for histopathologic examination.

### Immunohistochemistry for detection of RESTV antigen in tissues

For immunohistochemistry (IHC), five μm paraffin tissue sections were quenched for ten minutes in aqueous 3% hydrogen peroxide. Epitopes were then retrieved using Dako Target Retrieval solution (Dako, USA) in a BioCare Medical Decloaking Chamber. The primary antibody applied to the sections was a monoclonal anti-Reston NP (clone ABL-9) antibody developed and provided by the Public Health Agency of Canada. It was used at a 1:400 dilution for thirty minutes. An alkaline phosphatase labeled polymer, Mach 4 universal system® (BioCare Medical, USA) with the chromogen Vulcan Fast Red (VFR) (BioCare Medical, USA) for visualization were used. The sections were then counter-stained with Gill’s hematoxylin.

### In situ* hybridization for detection of RESTV in tissue sections*

For in situ hybridization (ISH) to detect viral genomic material, five μm paraffin-embedded formalin fixed tissue sections were cut, air dried then melted on to charged slides in a 60 °C oven. The slides were then cleared and dehydrated in xylene and 100% ethanol, then air dried. The sections were quenched for ten minutes in aqueous H_2_O_2_, boiled in target retrieval solution for fifteen minutes, rinsed in 100% ethanol and air-dried again. Then a final pre-treatment of protease plus enzyme for fifteen minutes at 40 °C was applied. The probe (V-RESTV-NP-C1, Advanced Cell Diagnostics) was applied and incubated at 40 °C for two hours. The hybridization amplification steps (AMP 1-6) were applied to the slides for the recommended times and temperatures as per the manual for the RNAscope® 2.5HD Detection Reagent—Red kit (Advanced Cell Diagnostics). The signal was then visualized by the chromogen Fast Red. The sections were then counter-stained with Gill’s No. 1 hematoxylin, dried and cover-slipped.

### *Combination immunohistochemistry and *in situ* hybridization staining for the identification of RESTV-infected macrophages and pneumocytes in lung tissue sections*

Five μm paraffin-embedded formalin fixed tissue sections were cut, air dried, then melted on to charged slides in a 60 °C oven. The slides were then cleared and dehydrated in xylene and 100% ethanol and allowed to air dry. Sections were quenched for ten minutes in aqueous hydrogen peroxide.

For identification of RESTV infected macrophages, epitopes were retrieved using an AR10 target retrieval kit (Biogenex, USA) in a BioCare Medical Decloaking Chamber. Mac387, a mouse anti-human macrophage monoclonal antibody (BioRad) was used at a 1:50 dilution at 4 °C overnight in a humidified chamber. Slides were then fixed with 10% neutral buffered formalin, rinsed, and followed by a final pre-treatment of protease plus enzyme for fifteen minutes at 40 °C. The RESTV-specific in situ hybridization probe (V-RESTV-NP-C1, Advanced Cell Diagnostics) was applied and incubated at 40 °C for two hours. RNAscope® 2.5HD Detection Reagent—Red kit (Advanced Cell Diagnostics) hybridization amplification steps (AMP 1-6) were followed per the kit instructions. The signal was then visualized by the chromogen Fast Red. Slides were treated with Co-detection blocker (Advanced Cell Diagnostics) and the IHC process was completed using a horseradish peroxidase labelled polymer and Envision® + system (anti-mouse) (Agilent Technologies), followed by reaction with the chromogen diaminobenzidine (DAB). The sections were then counter-stained with Gill’s No. 1 hematoxylin, dried and cover-slipped.

For the identification of RESTV infected pneumocytes, epitopes were retrieved using Co-Detection Target Retrieval (Advanced Cell Diagnostics, pH 9–10) for fifteen minutes. A 1:50 dilution of a mouse anti-human cytokeratin monoclonal antibody (clonesAE1/AE3, Agilent) was adsorbed overnight in a humidified chamber at 4 °C. The processes described for infected macrophage identification were followed, beginning with fixation of the slides with 10% neutral buffered formalin.

### RESTV RNA detection by quantitative real-time PCR

The following samples were tested for the presence of viral RNA by real-time reverse transcriptase polymerase chain reaction (rRT-PCR) targeting the RESTV L gene: citrated whole blood, nasal swabs, rectal swabs, oral swabs, nasal wash, urine, vitreous fluid, cerebrospinal fluid, bronchoalveolar lavage fluid (BALF), and group oral fluid. Fresh tissues collected at necropsy were cut into 0.1–0.5 g portions, distributed into tissue grinding tubes (Precellys Lysing Kit), and immediately frozen at  − 70 °C for further processing.

Tubes were later thawed, D-PBS added to 10%, and homogenization proceeded using a bead mill homogenizer for a minimum of forty-five seconds at the maximum setting. Each tube was centrifuged at 1500 × *g* for twenty minutes at 4 °C prior to sampling. Tissues, all swabs, BALF supernatant, BALF cell pellet, nasal wash fluid, CSF, vitreous, and urine were inactivated as a 1:10 suspension in Tripure Reagent (Roche), removed from the CL4 laboratory, and stored at  − 70 °C until further processing. Citrated blood samples were inactivated with TriZol LS (Invitrogen) per the manufacturer’s instructions prior to removal from the CL4 laboratory.

The MagMax CORE Nucleic Acid Purification Kit (Applied Biosystems, A73202) was utilized for extraction of total RNA from inactivated samples per manufacturer’s recommendation with some modification. Tripure Reagent or TriZol LS were utilized in place of the manufacturer’s lysis buffer for inactivation and removal of RESTV RNA from the CL4 laboratory. 650 µL of inactivated sample and 350 µL of kit-provided core binding buffer was utilized followed by a final elution volume of 30 μL using the automated MagMax Express 96 system (ThermoFisher Scientific). Enteroviral armoured RNA (ARM-ENTERO; Asuragen) was utilized as an exogenous extraction and reaction control. A standard curve was generated by serial tenfold dilutions of RESTV L-gene plasmid (Genescript) and included in every run allowing for the semi-quantification of viral load, measured in copies/reaction. Tripure-inactivated cell culture-derived RESTV stock virus was utilized in each run as a positive control. Extracted product was stored at  − 70 °C until real-time rRT-PCR was performed using a 4X TaqMan FAST Virus 1 Step kit, with the following primers and probes at a final reaction volume of 20 μL:

RESTV:

- RESTV L-gene Forward: 5’-GGA AGC GAG TCA ACC TTAG-3’; final concentration of 0.4 μM.
- RESTV L-gene Reverse: 5’-CGG GCT GTA TTG GTC GTT AT-3’; final concentration of 0.4 μM.
- RESTV L-gene Probe: FAM-5’-TCA GTG AAG/ZEN/TCC TGC AAA TGA CAC CA-3’-IABKFQ (purchased from IDT); final concentration of 0.1 μM.

ARM-ENTERO:

- ARM-ENTERO-31 Forward: 5’-ATG CGG CTA ATC CCA ACCT-3’; final concentration of 0.2 μM.
- ARM-ENTERO-31 Reverse: 5’-CGT TAC GAC AGG CCA ATC ACT-3’; final concentration of 0.2 μM.
- ARM-ENTERO-31 Probe: 5’-CAG GTG GTC ACA AAC-3’; final concentration of 0.06 μM.

Reactions were run on a 7500 Fast machine (Applied Biosystems) with the following conditions: 50 °C for five minutes; 95 °C for twenty seconds; 40 cycles of 95 °C for three seconds followed by 60 °C for thirty seconds. Ct values were determined by the 7500 Fast software; and the respective virus gene copy numbers were calculated for each sample, based on Ct values for each respective standard curve. All samples were assayed as technical replicates.

Sample baselines were determined by the default settings of the ABI 7500 software. The Ct value threshold was determined by separate analysis of the standard curve, and then the threshold applied to the remainder of the samples to determine the Ct values.

### Virus isolation from samples and tissues

Isolation was conducted on Vero E6 cells plated in a forty-eight-well format at 80% confluence. Media was removed and monolayers were washed with D-PBS. Samples were homogenized as described above for RNA detection and diluted in DMEM, 1:10 and 1:100 for non-tissue samples and 1:10 to 1:100,000 for tissues, and adsorbed to washed monolayers for one hour at 37 °C and 5% CO_2_. DMEM with 2% FBS, penicillin/streptomycin, and L-glutamine was then added to each well and plates were returned to humidified incubation at 37 °C and 5% CO_2_. All plates were read for cytopathic effect at day fourteen for evidence of virus replication. Samples were assayed in duplicate and the sample origin was blinded until after results were recorded. Duplicate negative controls (DMEM) and positive controls (challenge virus stock) were included on each test plate. Tissue homogenates and samples positive for CPE were confirmed by immunocytostaining as described above for virus titration. The 1:100 dilution was used for non-tissue samples and the lowest dilution that was suspect positive was use for tissue homogenates. The samples were not blinded for confirmation.

### Antibody analyses

RESTV-specific IgM and IgG were measured in serum by enzyme-linked immunosorbent assay (ELISA) as described by Haddock et al.^10^ using commercially available, recombinant RESTV glycoprotein without the transmembrane component (rRESTV GPdTM) (IBT Bioservices; 0504-015) and peroxidase-labeled rabbit anti-pig IgG (polyclonal, H + L, 1:1000 dilution; Invitrogen; PA1-28602) or goat anti-pig IgM (polyclonal, 1:5000 dilution; BioRad laboratories; AA148) with a two-step substrate system (KPL ABTS Peroxidase Substrate System; SeraCare). RESTV-specific IgA was measured in nasal wash fluid or oral fluid collected by rope chew using an adaptation of the IgM and IgG protocol. The same rRESTV GPdTM antigen was diluted in PBS (1:5000) and used to coat Nunc MaxiSorp F plates at 0.5 µg/mL; 5% skim milk and 0.5% Tween-20 in PBS was used as both a washing and blocking buffer. Serial two-fold dilutions made in blocking buffer were assayed in duplicate beginning at a 1:4 dilution until end-point. Bound antibody was detected with peroxidase-labeled goat anti-pig IgA (polyclonal, 1:5000 dilution; BioRad Laboratories; AA140) followed by the ABTS substrate described for the IgM and IgG ELISAs. Absorbance for all three ELISAs was measured at 405 nm using a plate reader (BioTek Epoch microplate spectrophotometer with Gen5 Data Analysis software, version 1.11). Sera with an OD greater than the average negative OD plus three standard deviations were considered positive.

### Viral neutralization assay

Neutralization activity of all serum collected during the study were determined by a modified plaque reduction neutralization test (PRNT) against RESTV. Serial two-fold dilutions of heat inactivated (56 °C for thirty minutes) sera were incubated with fifty pfu total of virus for 1 h at 37 °C. Each mixture of serum and virus was then added to duplicate wells containing confluent monolayers of Vero E6 cells in a ninety-six-well format, incubated for one hour at 37 °C, and overlaid with 150 μl of 1.75% carboxymethylcellulose (CMC) in DMEM per well. Plates were then incubated at 37 °C and 5% CO_2_ for seventy-two hours and fixed with 10% buffered formalin. Virus not neutralized by the serum was measured by immunocytochemistry as described for the titration of virus. Serum dilutions resulting in > 70% reduction of plaque counts compared to virus controls were considered positive for virus neutralizing activity. Serum from each animal was screened for non-virus specific neutralizing activity against assay components.

### Genome sequencing

Challenge virus stock used as the inoculum for both cohorts one and two and, based on results from rRT-PCR, homogenized tissue samples collected from each animal were evaluated by whole genome sequencing. A database of all publicly available *Orthoebolavirus restonense* complete genomes was downloaded from NCBI on June 7, 2021. Genomes were aligned with MAFFT v7.48 on default settings and the resulting alignment was inspected within Geneious v9.1.8 in order to confirm that maximum sequence divergence did not exceed 5%, as per the instructions of the Primal Scheme website^24,25^. Additionally, Primal Scheme recommends that genomes with 99% or higher identity be removed in order to reduce bias during primer selection. In order to remove genomes with high similarity, CD-HIT-EST web server was used with a sequence identity cut-off of 0.99^26^. The resulting database was used to generate PCR primers for tiled whole genome amplification using the Primal Scheme web server^27^. Designed primers were manually compared to the previously generated whole genome database and inspected using Geneious v9.1.8 to look for mismatches within primers that could potentially impact amplification efficiency^25^. Based on this manual inspection, it was determined that the scheme with an amplicon size of 1150 bp yielded the best primers.

Reverse transcription was performed on extracted nucleic acid using the LunaScript RT SuperMix Kit (New England Biolabs Inc.) according to manufacturer’s instructions. PCR amplification was performed using the previously designed primers and Q5 High-Fidelity 2X Master Mix (New England Biolabs Inc.) according to the conditions used by Quick et al.^27^, except with thirty-two amplification cycles. Here, overlapping amplicons tiling across the genome were generated. In order to prevent the generation of unwanted PCR products, two separate reactions were generated for each sample containing primers for alternating PCR products. Following amplification, the two reactions for each sample were combined, cleaned using AMPure XP (Beckman Coulter) size selection beads at 1 × concentration, and quantified using Qubit 1 × dsDNA HS Assay Kit on the Qubit 3.0 Fluorometer (Thermo Fisher Scientific). Sequencing libraries were generated using the Nextera XT library preparation kit (Illumina) as per manufacturer’s instructions. Libraries were quantified, pooled and sequenced on the Illumina MiSeq using a V3 flow cell and a 600 cycle kit (Illumina).

### Supplementary Information

Supplementary Information 1.

Supplementary Information 2.

## Data Availability

The datasets generated during and/or analysed during the current study are available from the corresponding author on reasonable request.
