# Supplementary material for: Domestic pigs are susceptible to experimental infection with non-human primate-derived Reston virus without the need for adaptation
Source: Sci Rep. 2024 Jan 6;14:715. doi: 10.1038/s41598-024-51280-8 (PMC10771446; doi:10.1038/s41598-024-51280-8)
Supplement: Supplementary file 1 — Supplementary Information 1. [file 41598_2024_51280_MOESM1_ESM.docx]

**Domestic Pigs are Susceptible to Experimental Infection with Non-Human Primate-Derived Reston Virus Without the Need for Adaptation.**

Lewis *et al.* Supplement.

**
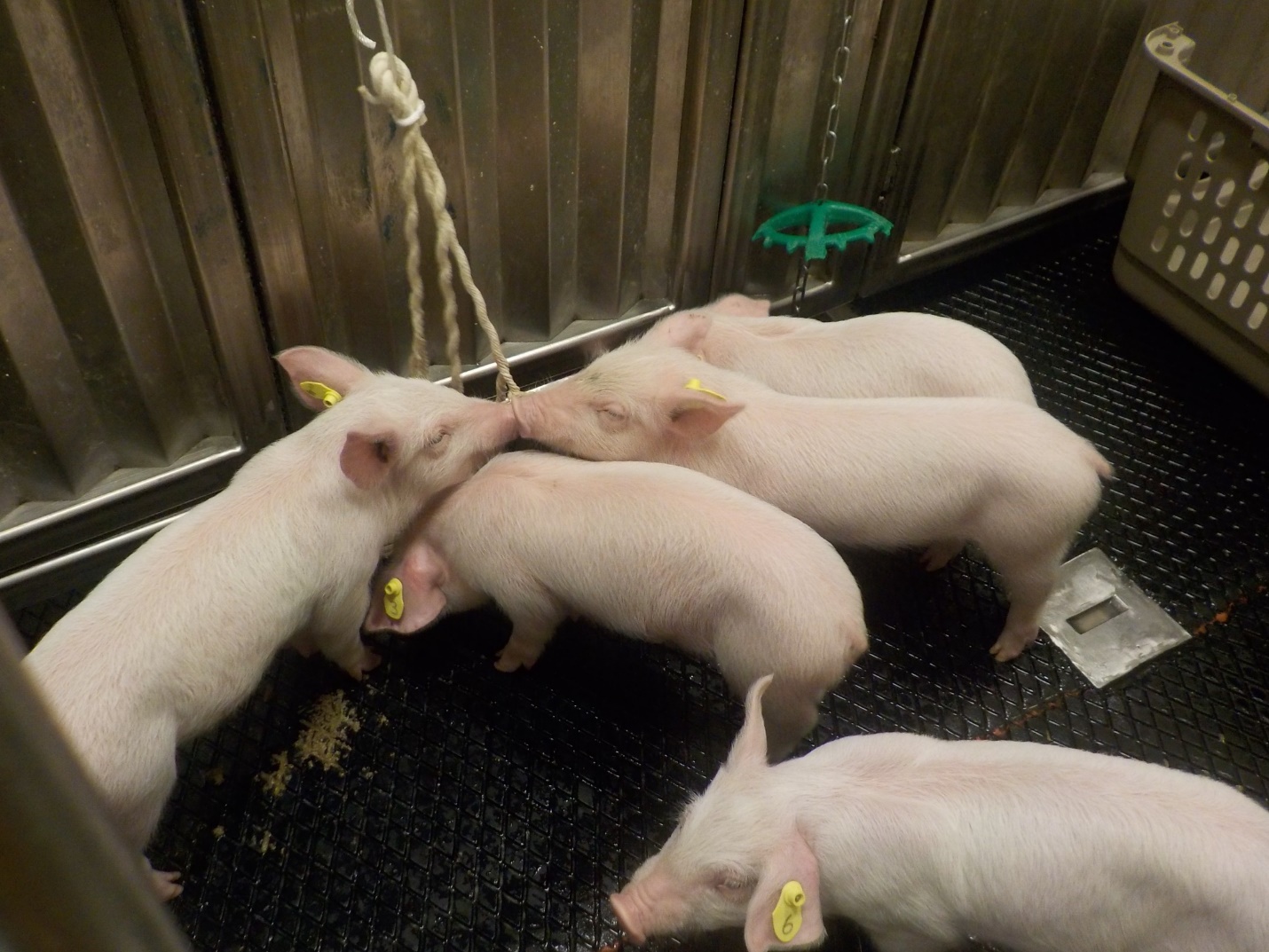
**

**Supplement Figure 1: Collection of group oral fluid samples.** Oral fluid (OF) samples were collected utilizing a cotton rope hung in the housing pen. As shown in the image, pigs readily interacted with the rope as a form of enrichment. After approximately twenty to thirty minutes, the rope was removed, placed in a sterile bag, and the OF wrung out of the rope. The OF was then transferred to a sterile container to be split for further testing.

a.

**
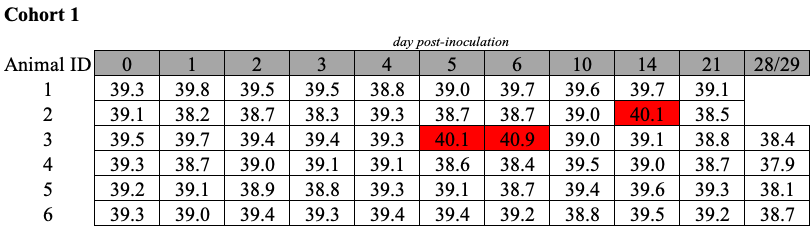
**

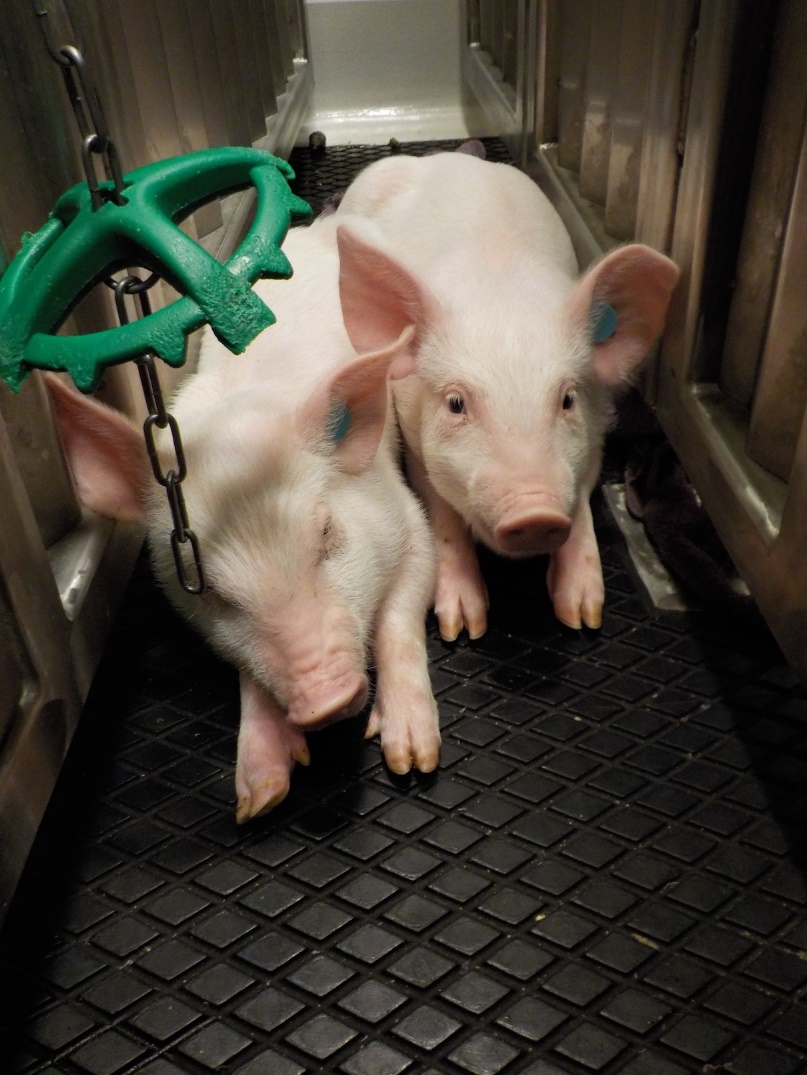
b. d.

**
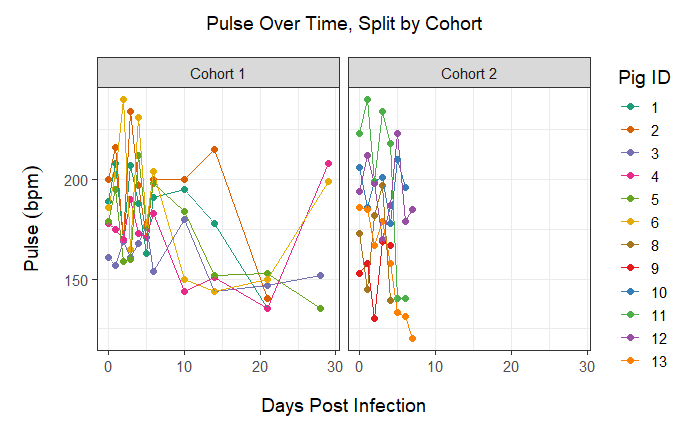
**

c.

**
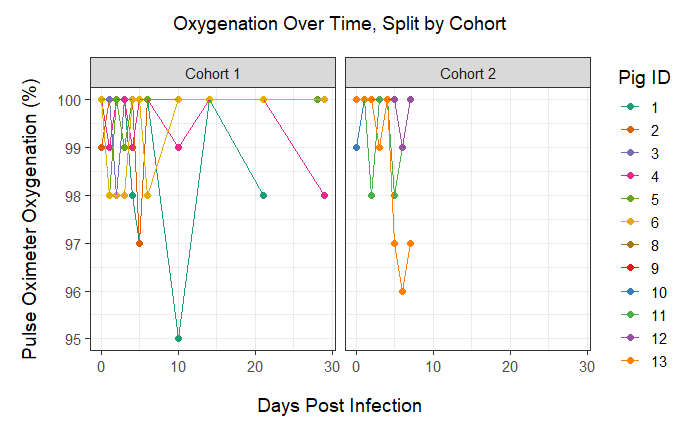
**

Pig 12

e.


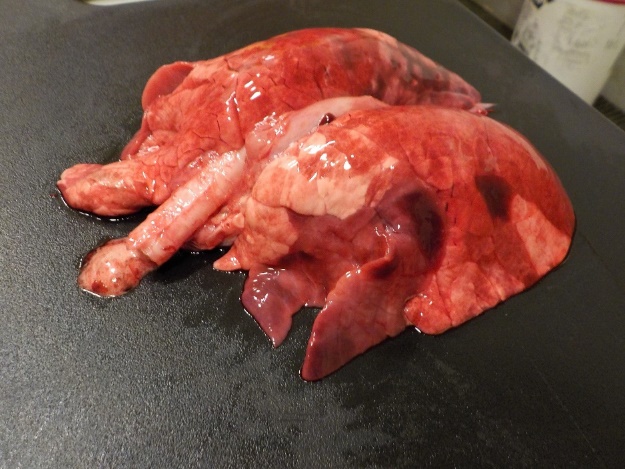


Pig 13

**Supplement Figure 2: Clinical features and parameters of Reston virus infection in domestic pigs.** Individual daily rectal temperatures from pigs experimentally infected with Reston virus (RESTV) are provided (a). Temperatures greater than 40.0^o^C, the study's designation for fever, are highlighted in red. No significant trends were noted in either pulse (b) or oxygen saturation (c) as collected by aural pulse oximetry in sedated animals. Of the eleven inoculated pigs, only pig 13 exhibited signs of clinical disease, which included severe respiratory distress (dyspnea, tachypnea with abdominal effort) and a reluctance to move (d). The image shows pig 13 (left) and pig 12 (right) at seven days post-infection (dpi). These pigs are shown temporarily corralled immediately before sedation at seven dpi. Pig 12 was quiet, alert, and responsive while pig 13 was physically depressed, reluctant to move, and demonstrating signs of respiratory distress (tachypnea and dyspnea with increased abdominal effort). During necropsy, pink to red, frothy fluid consistent with pulmonary edema was present in the trachea (e, arrow) and on cut section of the lung.

1. Complete blood counts


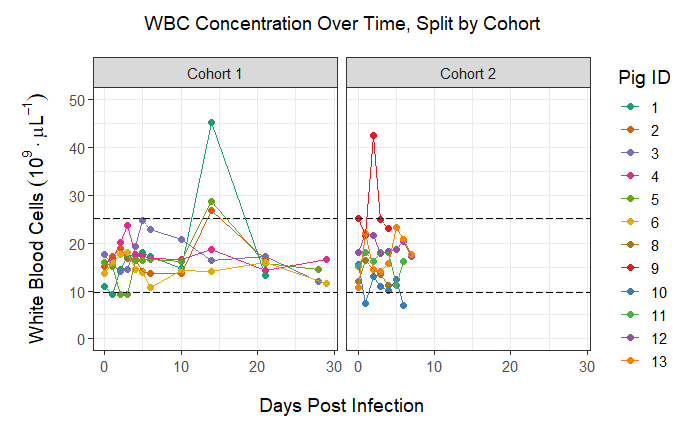

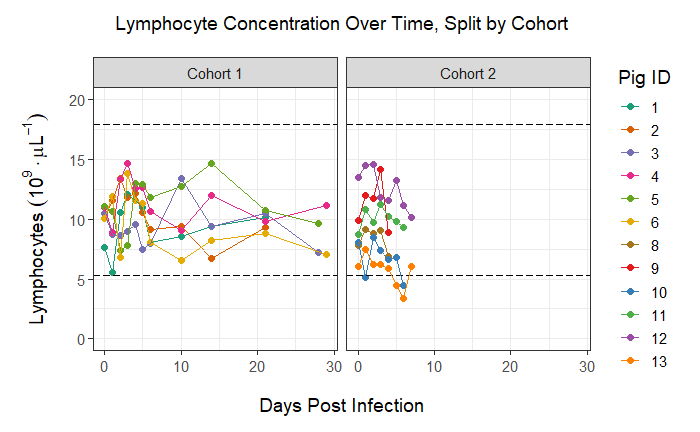

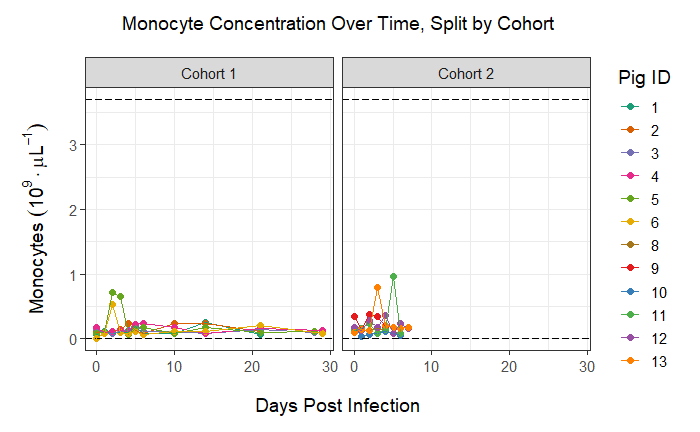

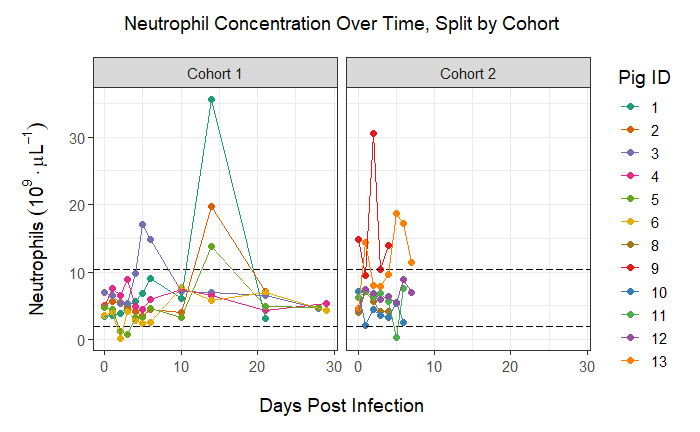


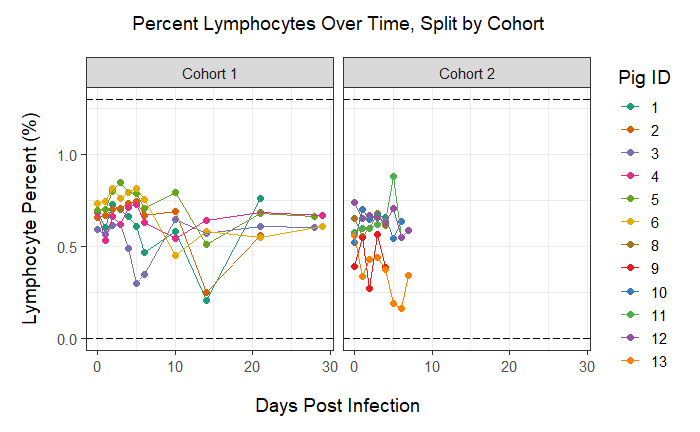

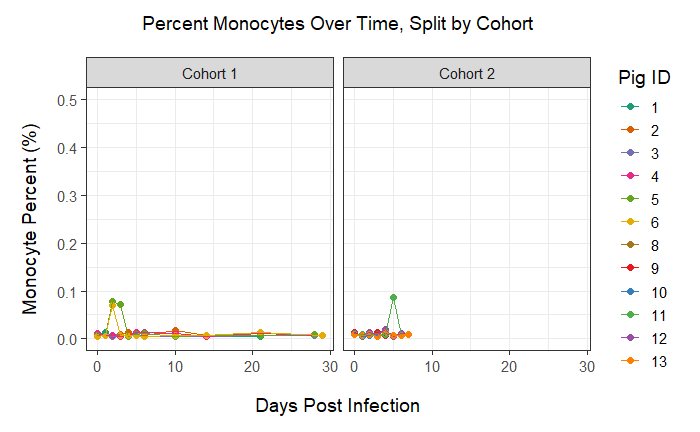

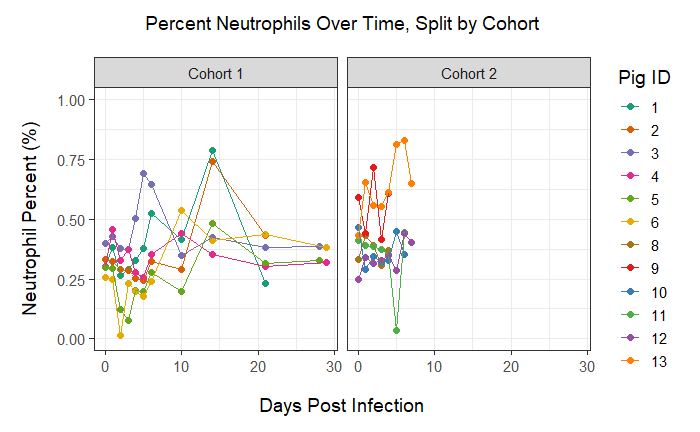

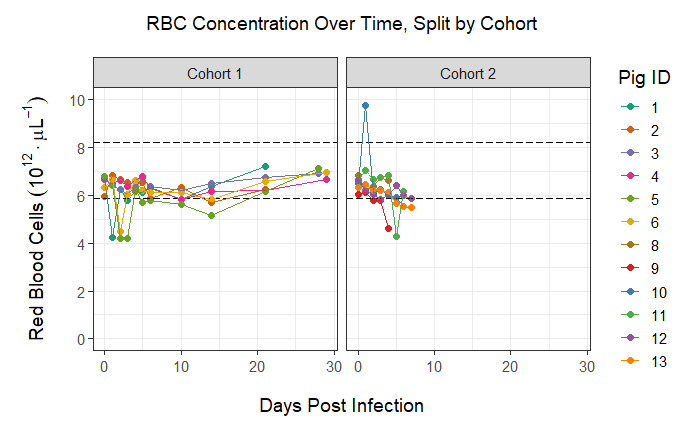


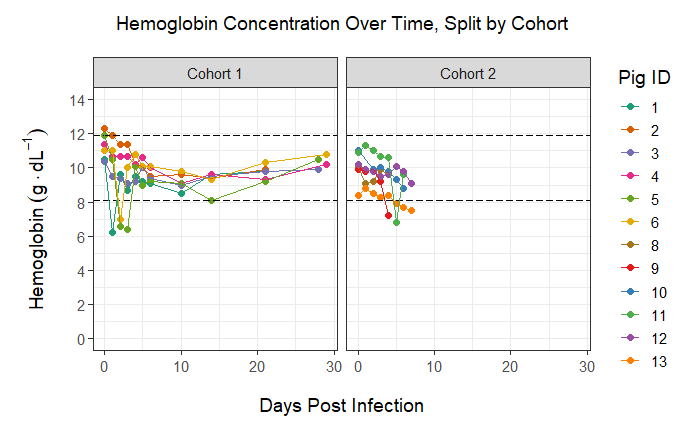

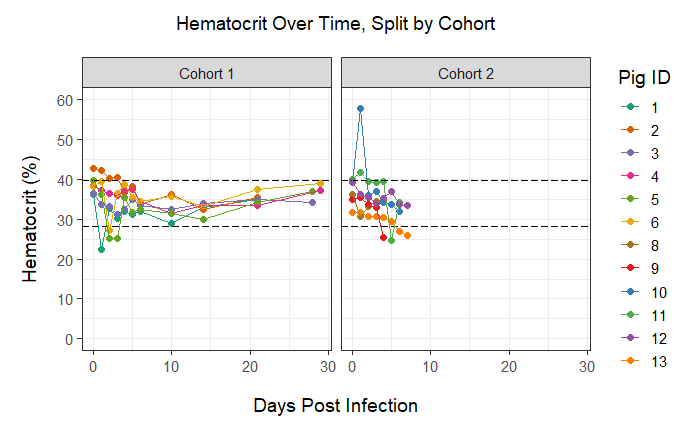

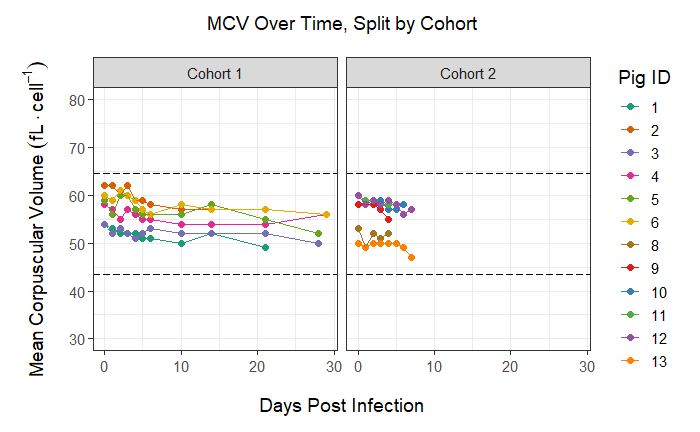

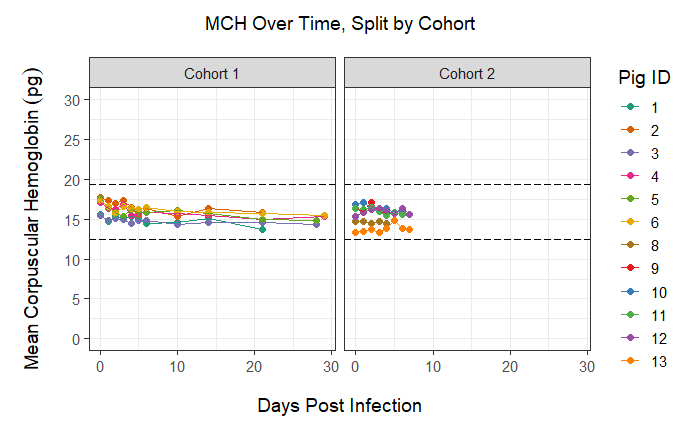


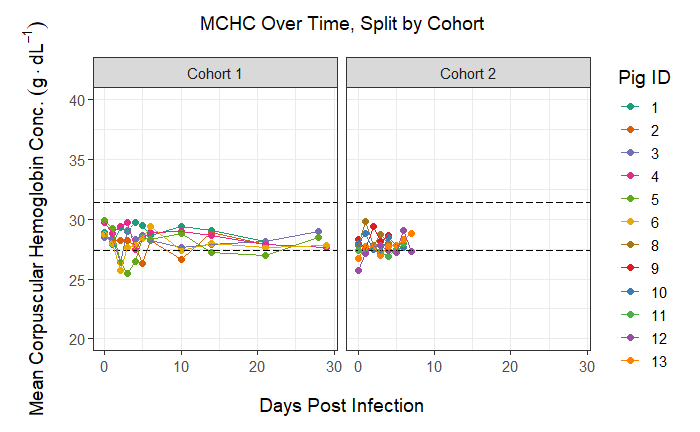

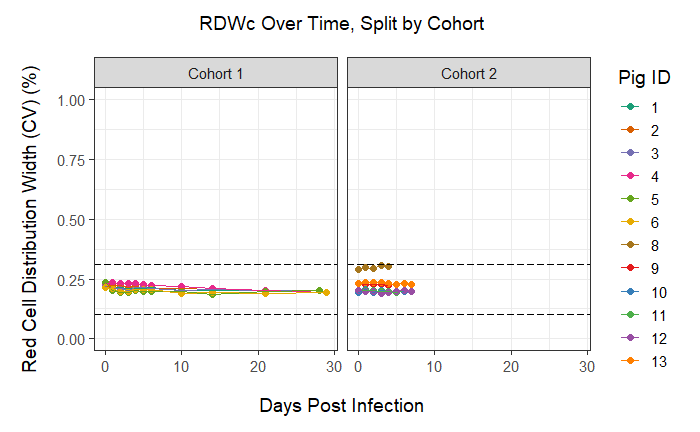

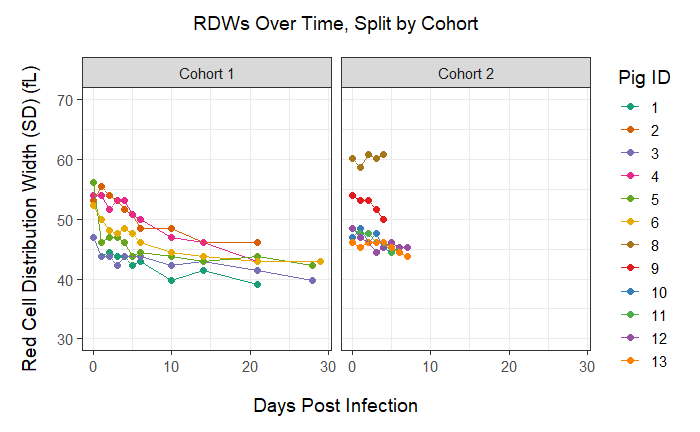

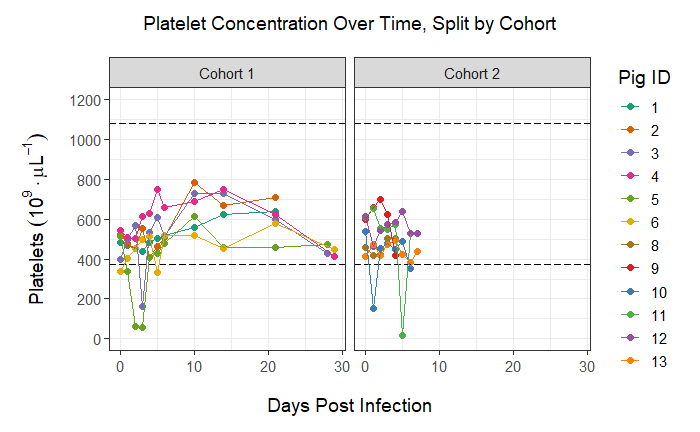


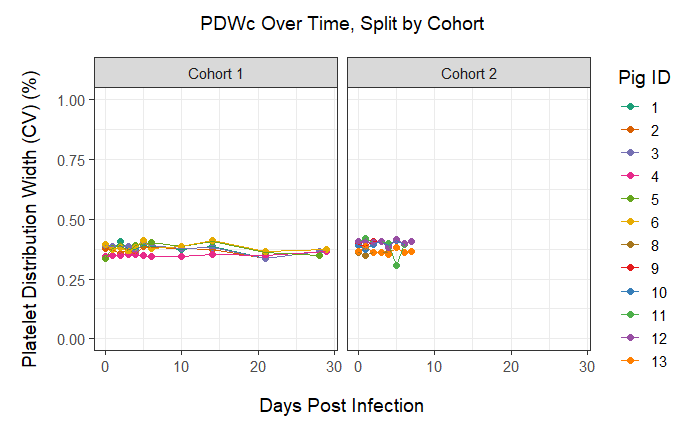

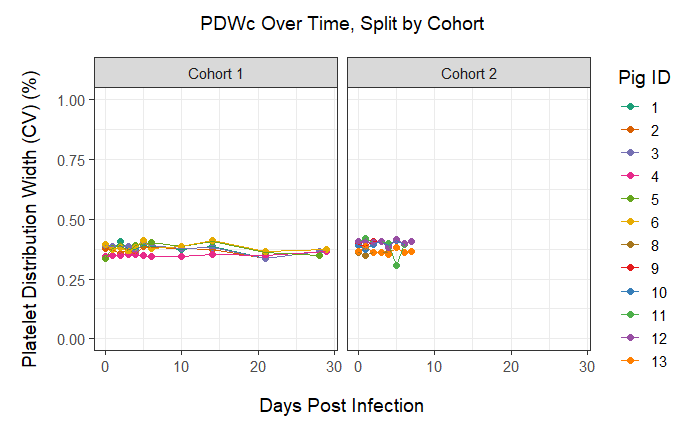

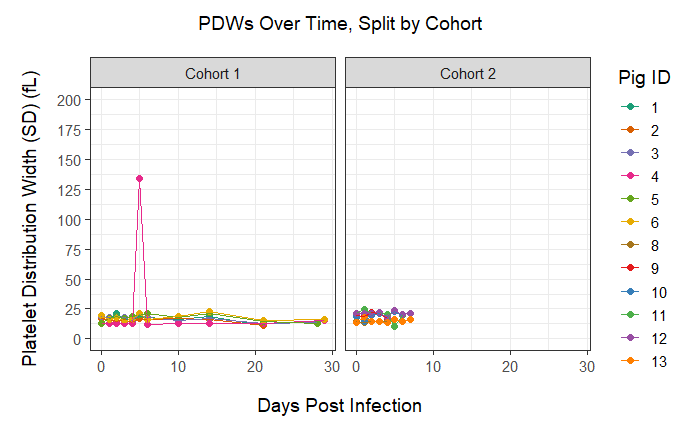

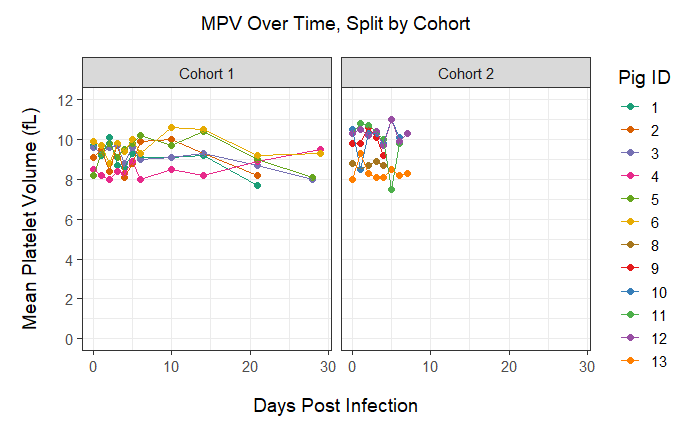


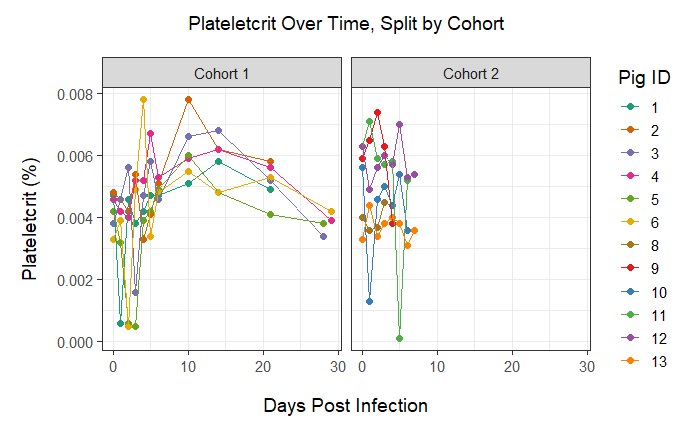


1. Blood chemistries


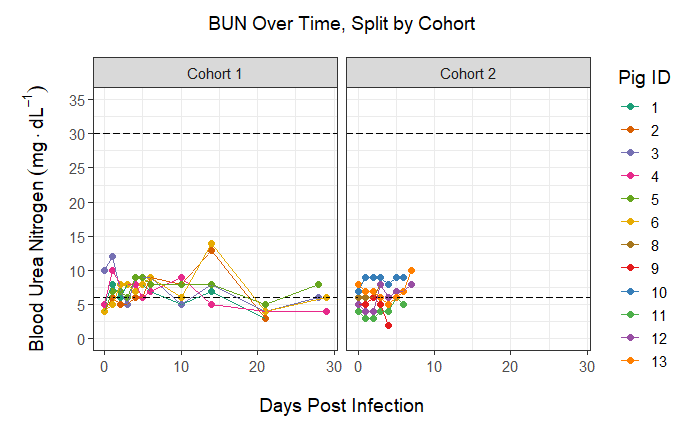

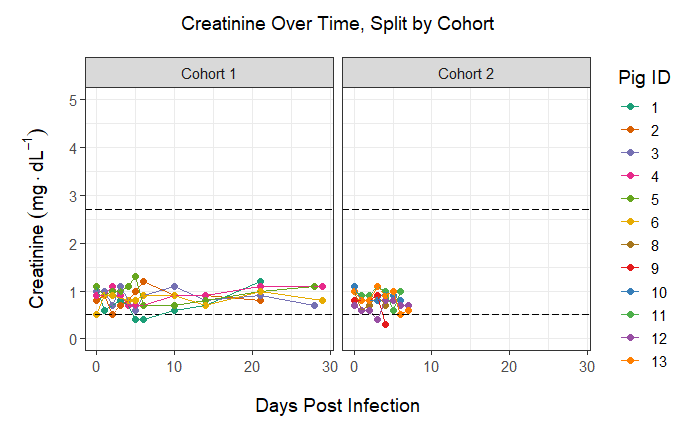

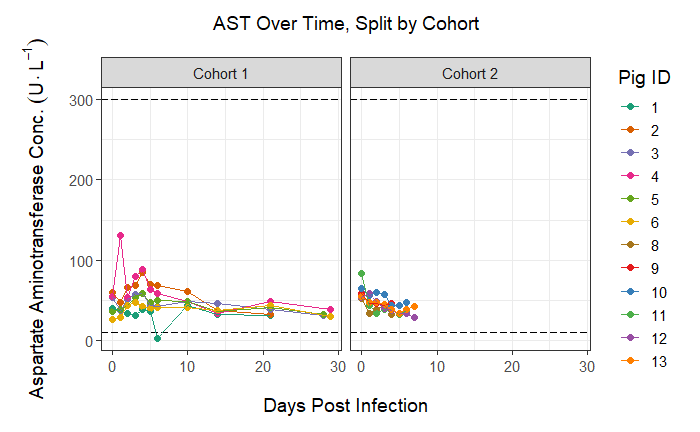

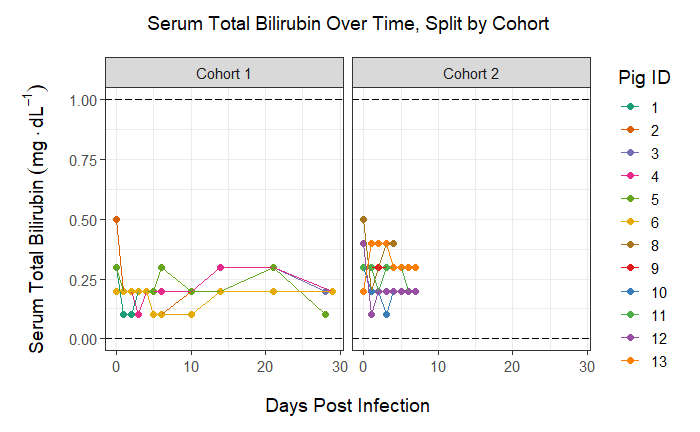


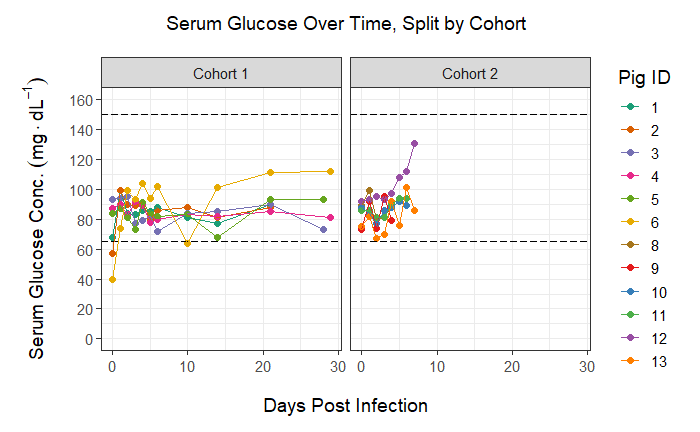

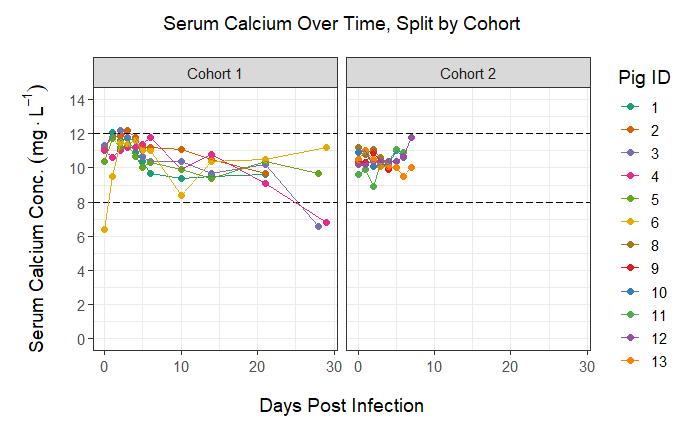

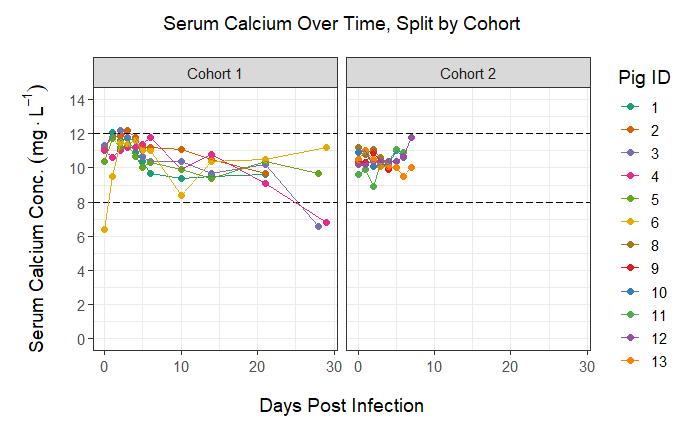

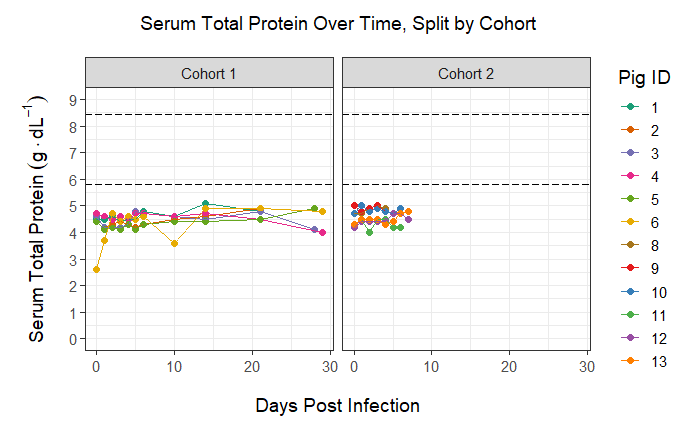


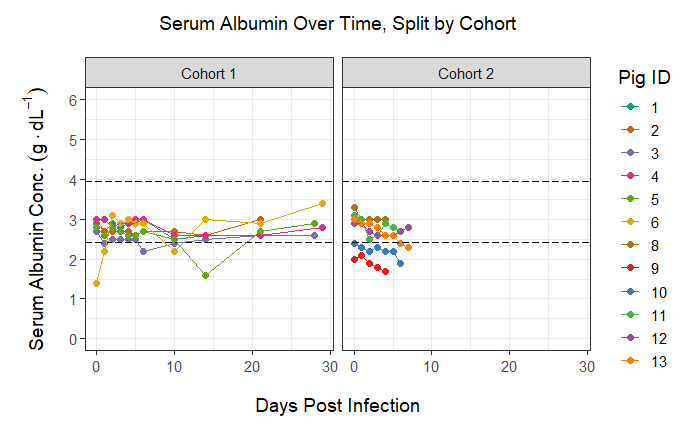

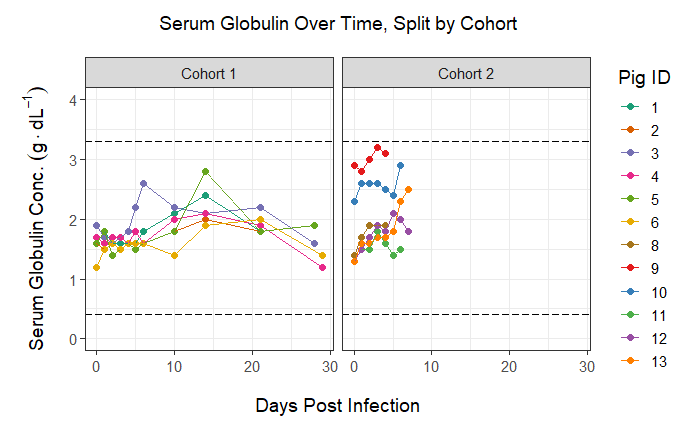

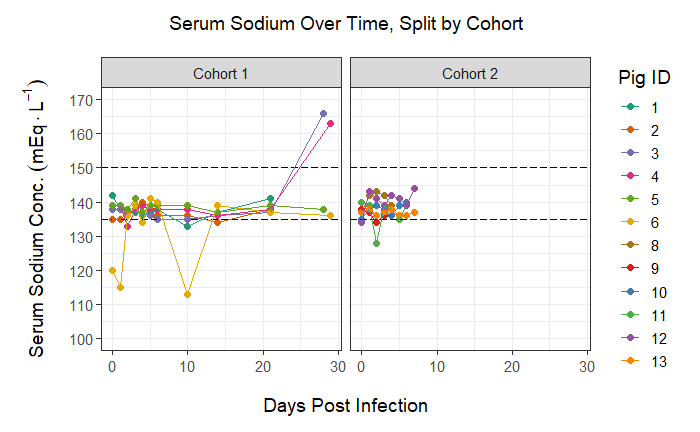

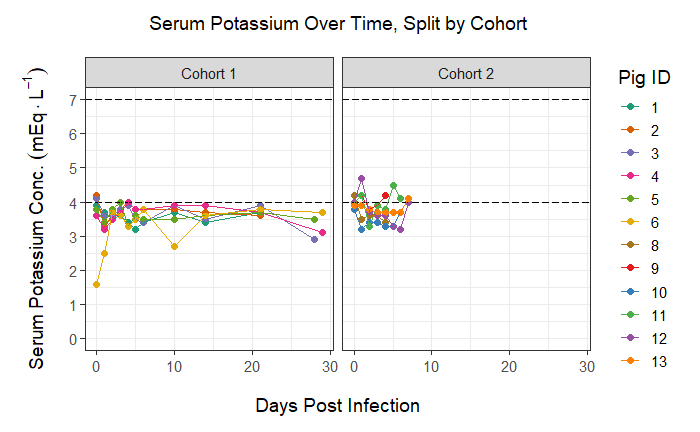


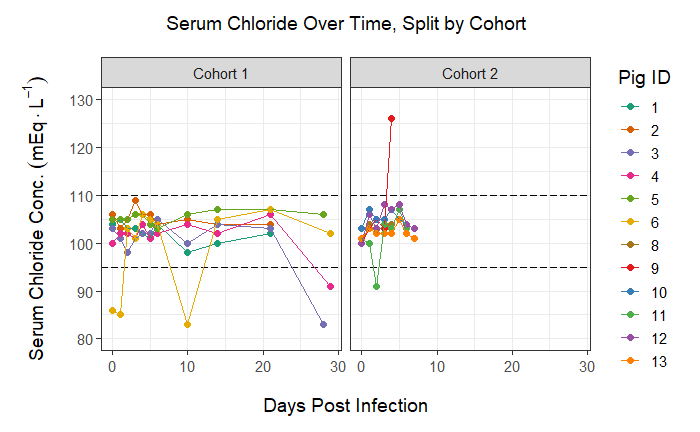

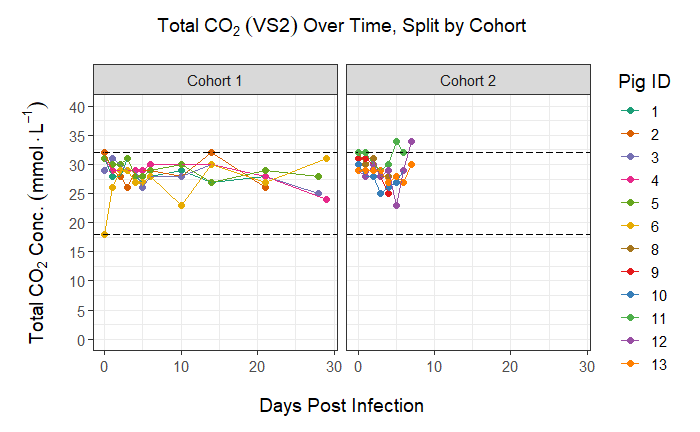


1. Blood gases


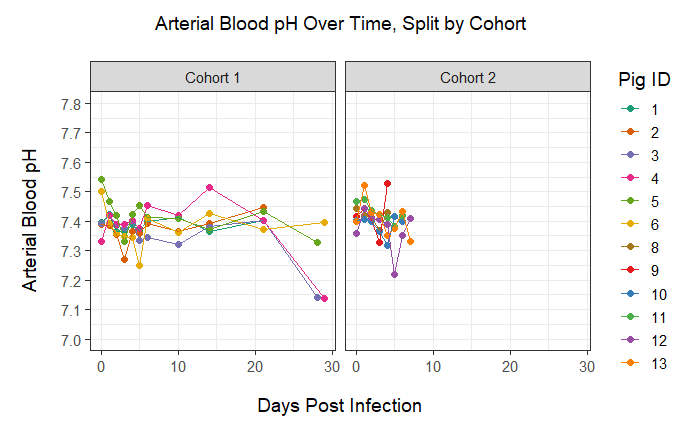

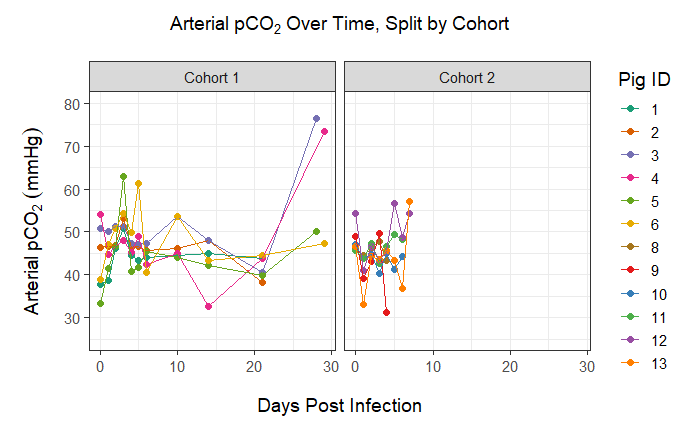

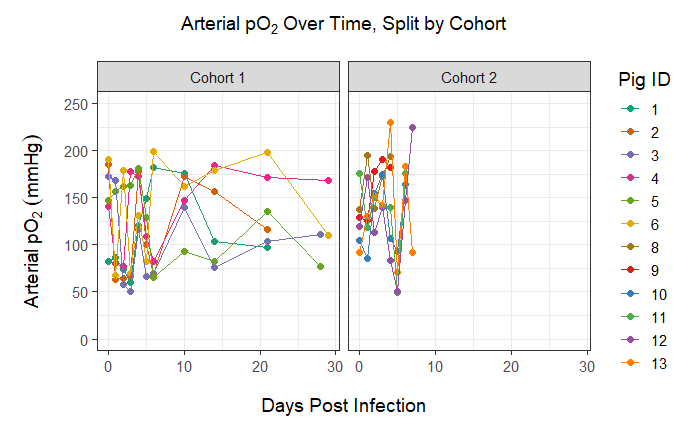

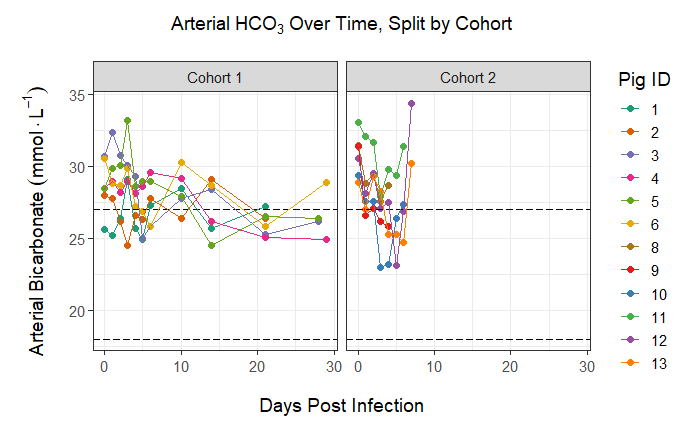

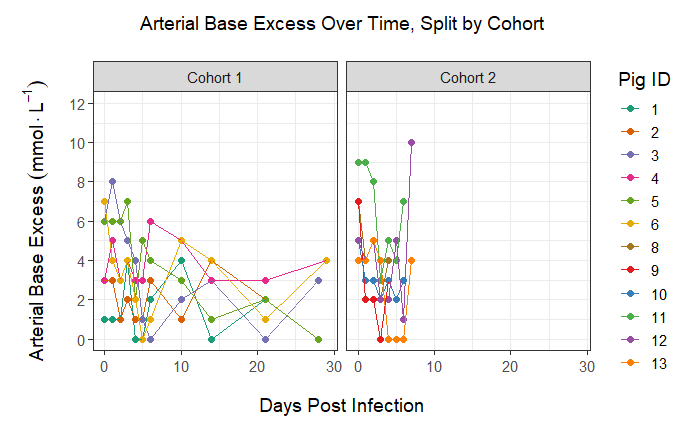

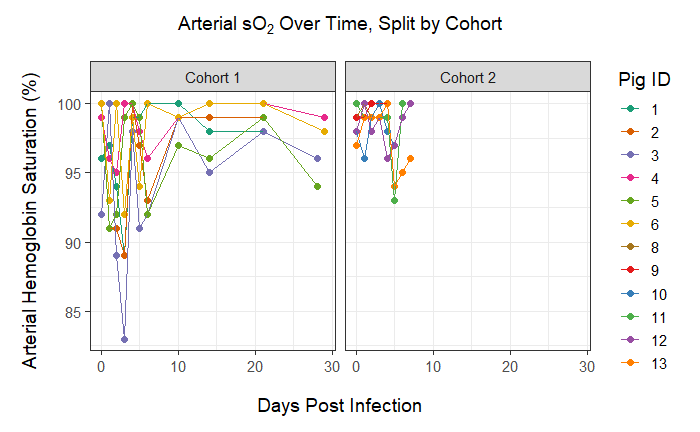

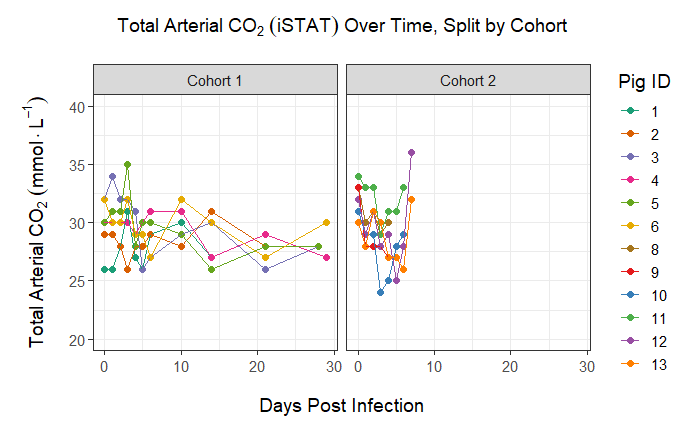

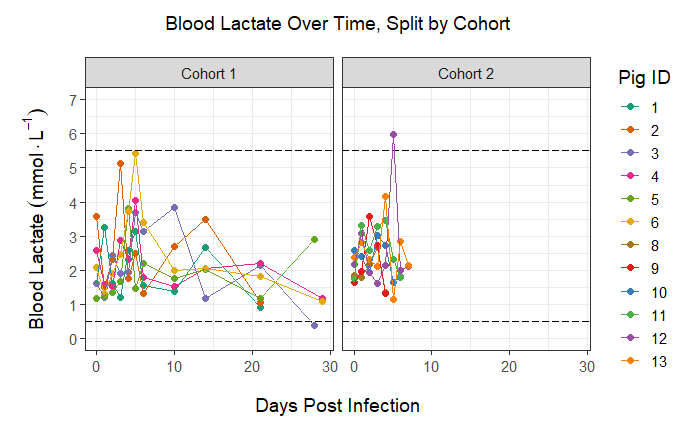


**Supplement Figure 3: Clinical pathology results from Reston virus infected pigs.** Blood was collected from pigs experimentally infected with Reston virus (RESTV) at the timepoints outlined in Figure 1. Clinical pathology analysis run on these samples included complete blood counts (a), blood chemistries (b), and blood gases (c). Dashed lines represent expected normal ranges. No significant trends were detected during the study.

a.
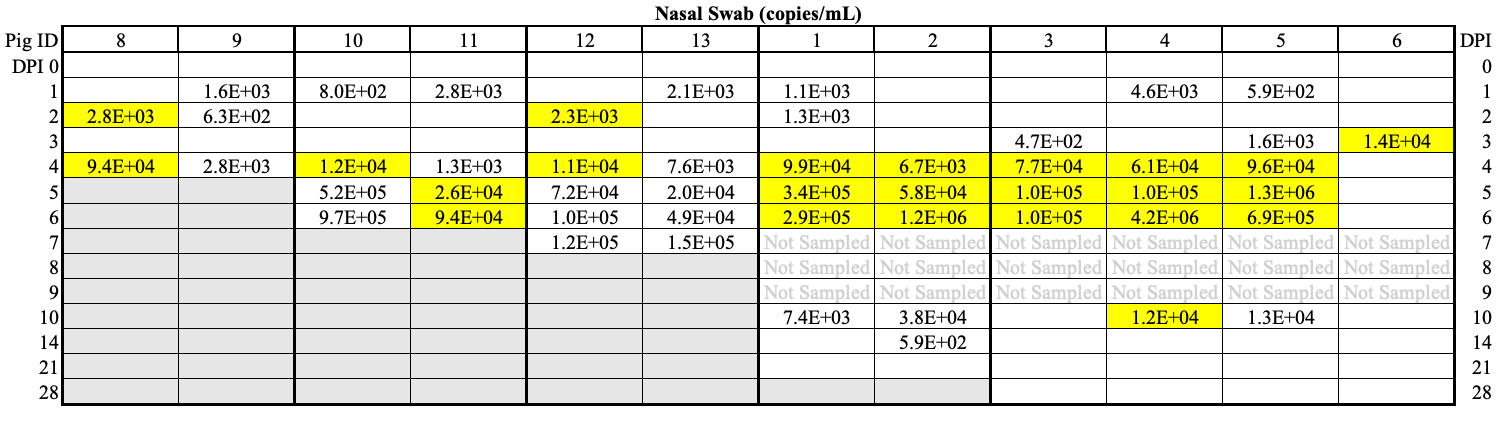


b.


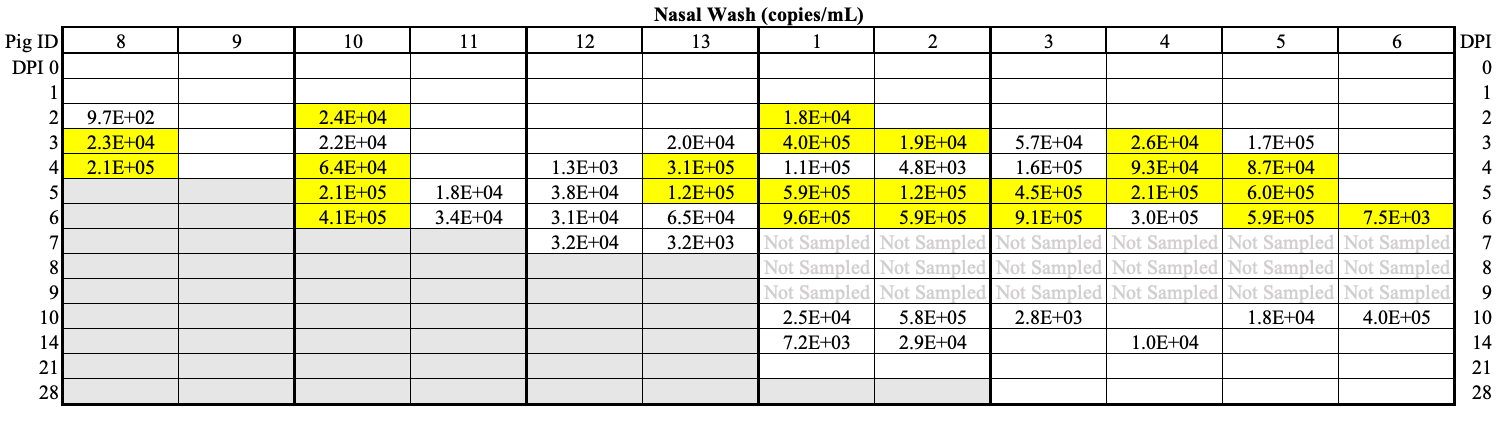


c.


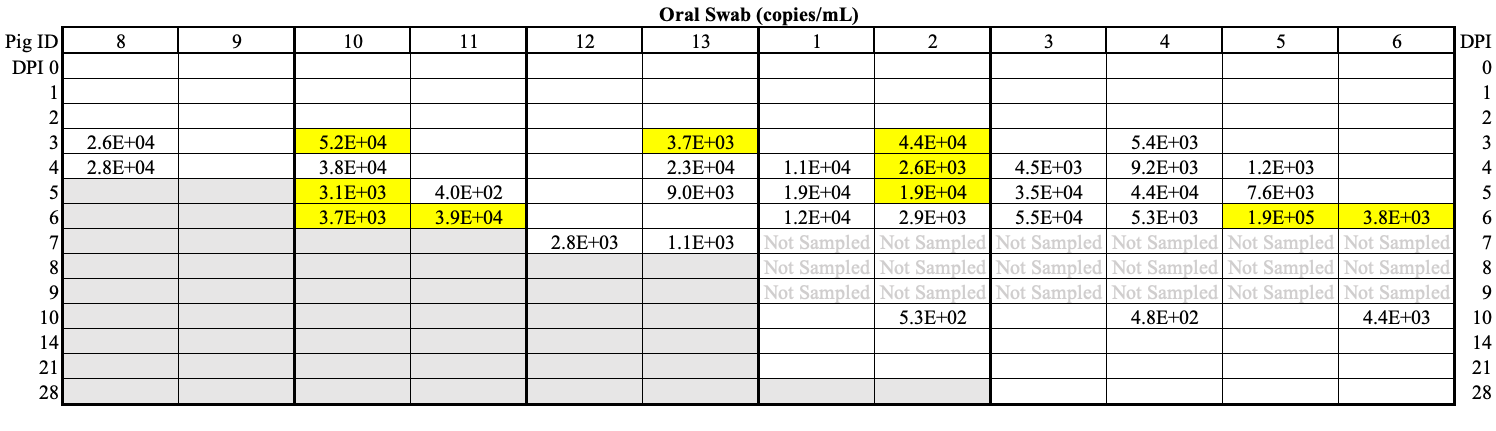


d.


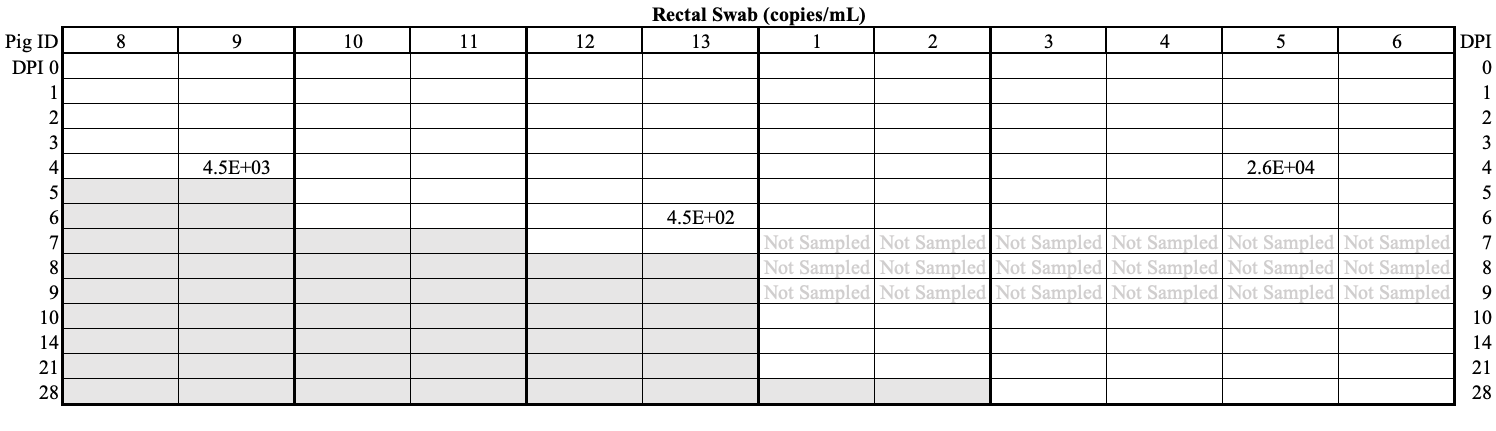


e.


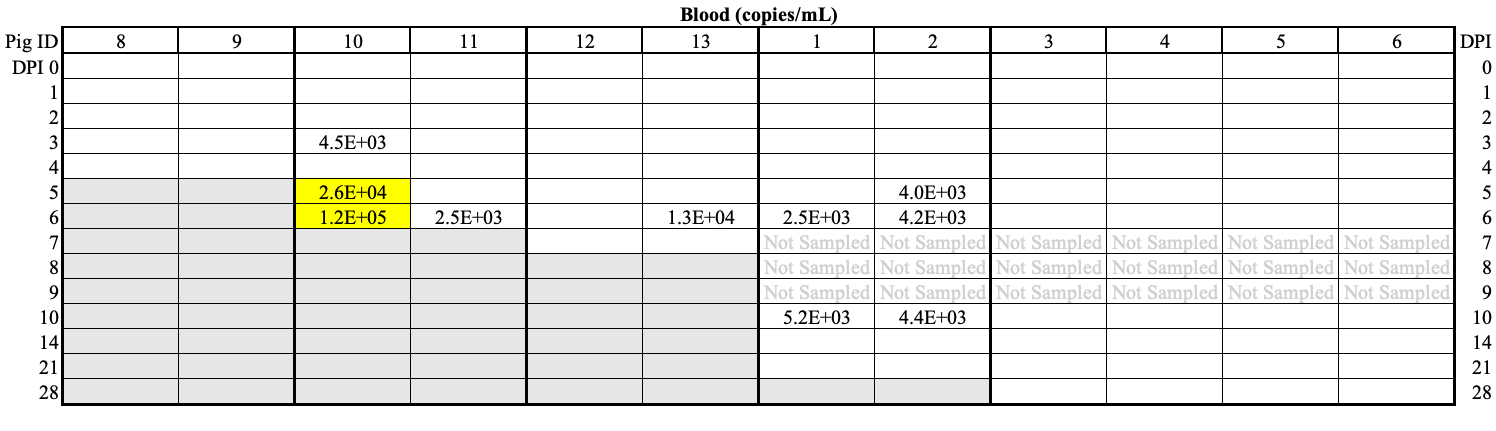


f.


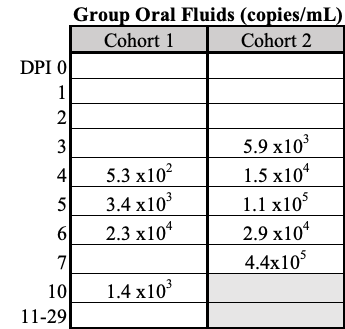


**Supplement Figure 4: Detection of Reston virus in individual samples from experimentally infected pigs.** Results are provided for each of the sampling methods utilized in the study. Values are reported as the mean Reston virus (RESTV) copies per mL from technical replicates as determined by rRT-PCR for each pig and each day post-infection (dpi) sampled. The yellow highlighted values represent samples in which infectious virus was recovered by isolation in cell culture. Samples include: nasal swab (a), nasal wash fluid (b), oral swab (c), rectal swab (d), blood (e), and group oral fluid (f) as collected by rope chew. dpi, day post-infection.

a.

**
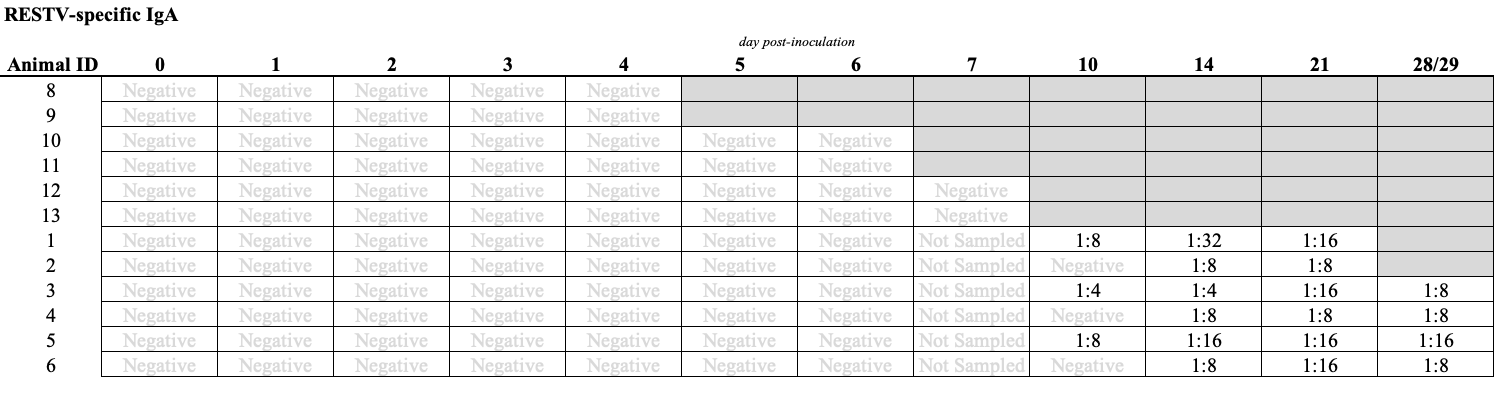
**

b.


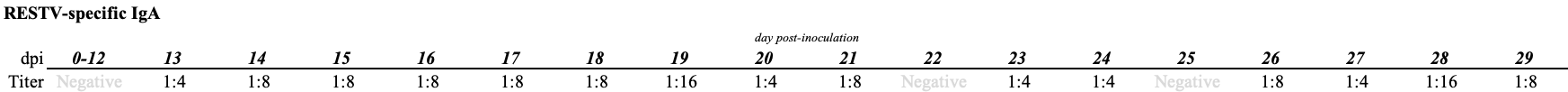


c.

**
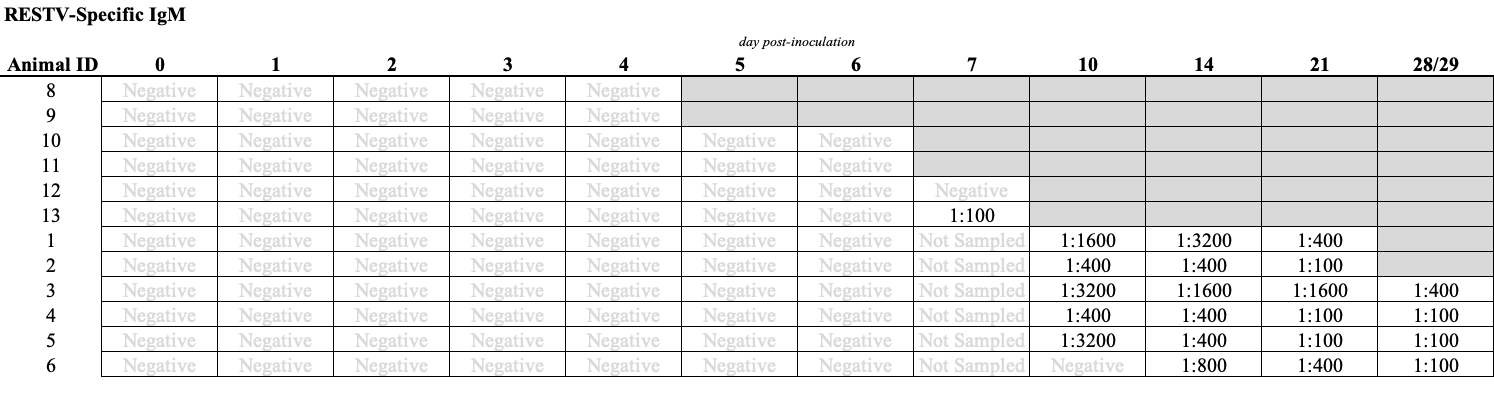
**

d.

**
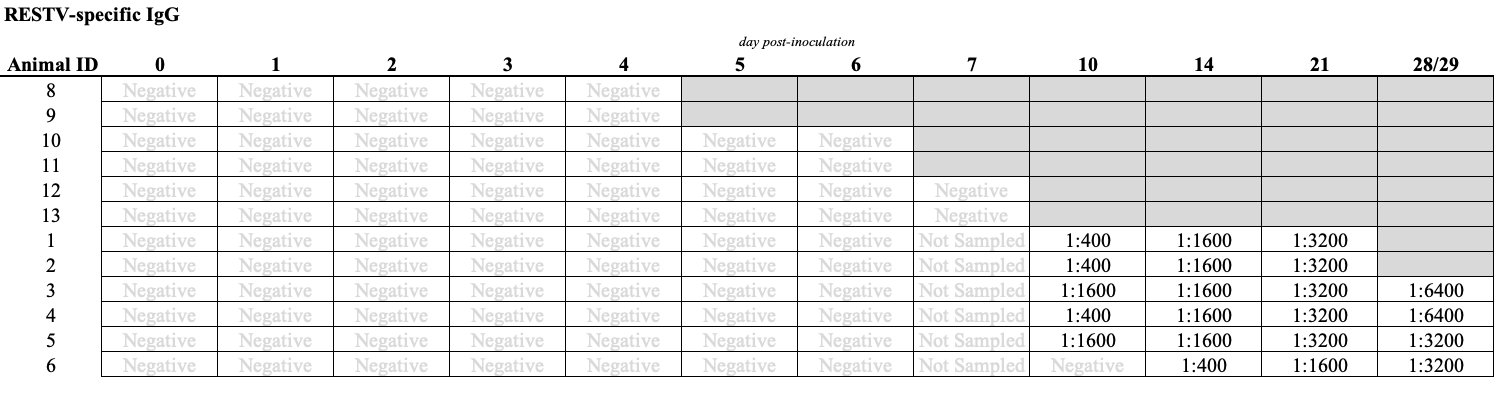
**

**Supplement Figure 5: Reston virus infected pigs develop a robust IgA, IgM, and IgG antibody response.** Results shown are titers for Reston virus (RESTV)-specific antibody as detected by enzyme-linked immunosorbent assay (ELISA) in experimentally infected pigs. IgA was detected in both individual animal nasal wash fluid (a) as well as group oral fluid collected by rope chew (b). Both IgM (c) and IgG (d) were detected in individual animal sera collected at the listed timepoints.

4 dpi 6 dpi 7 dpi

a. c. e.

HE


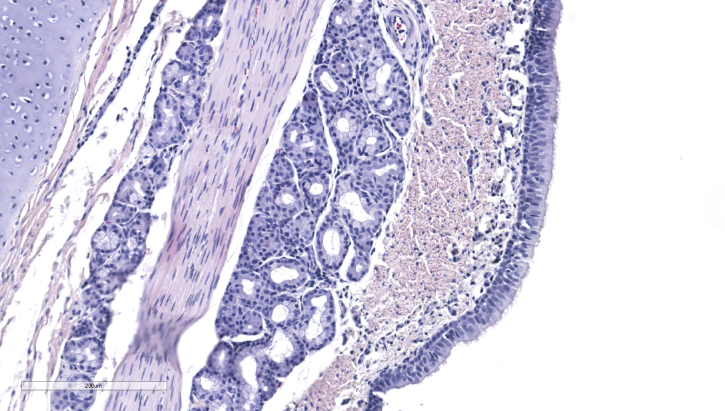

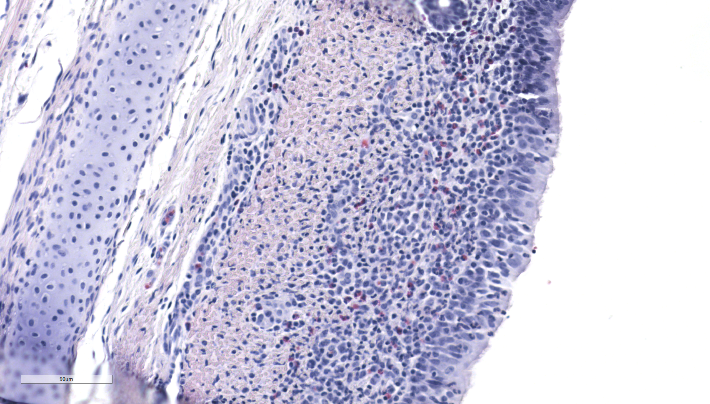

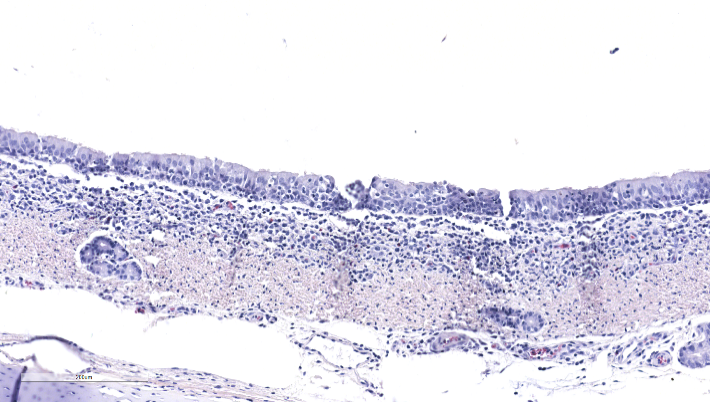


b. d. f.

IHC


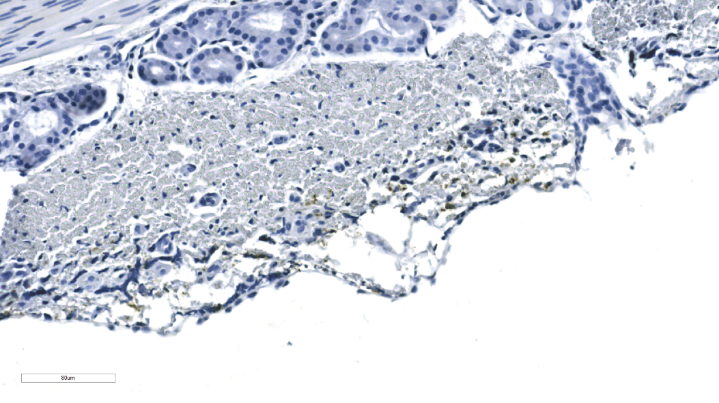

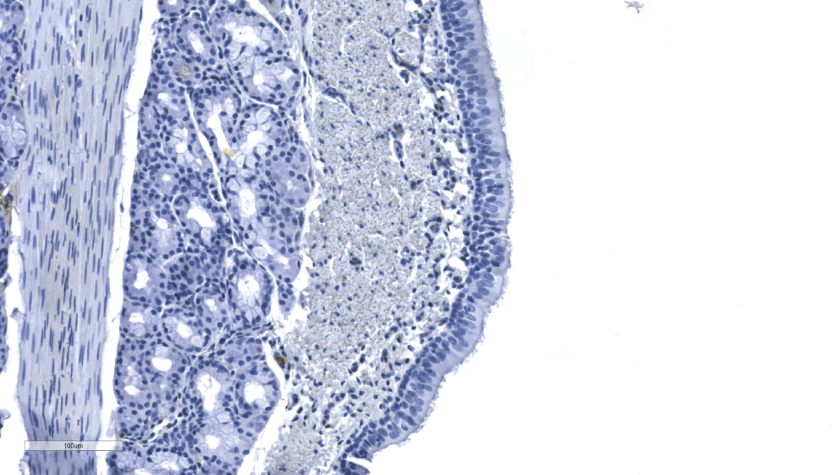

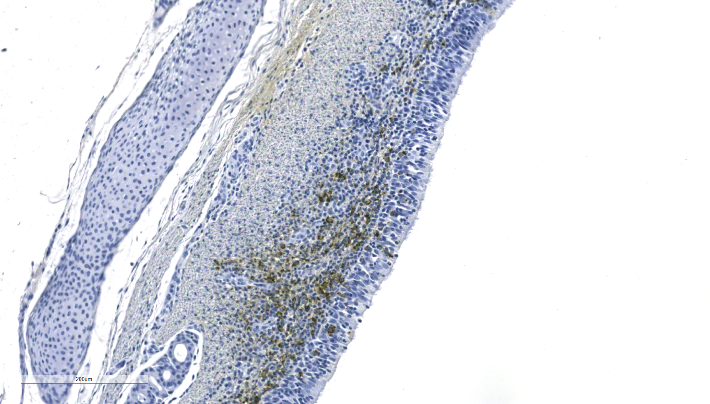


**Supplement Figure 6. Histopathology and immunohistochemistry findings in the trachea of Reston virus-infected pigs.** No histopathologic lesions (a) or antigen (b) were detected at four days post infection (dpi). At six dpi, there is mild submucosal inflammation (c) which is associated with the distribution of viral antigen detectable by IHC (d). Mild submucosal and epithelial inflammation was present at seven dpi (e) and there were a few small scattered areas where a small amount of antigen could be detected (f). Tissues originated from Pig 9 (a,b), Pig 10 (c,d), and Pig 13 (e,f).

4 dpi 6 dpi 7 dpi

a. c. e.

HE


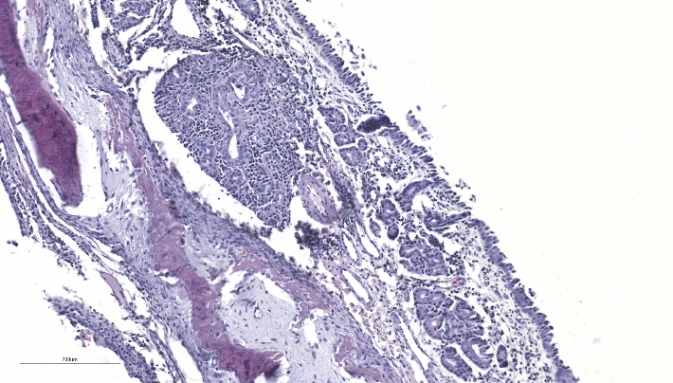

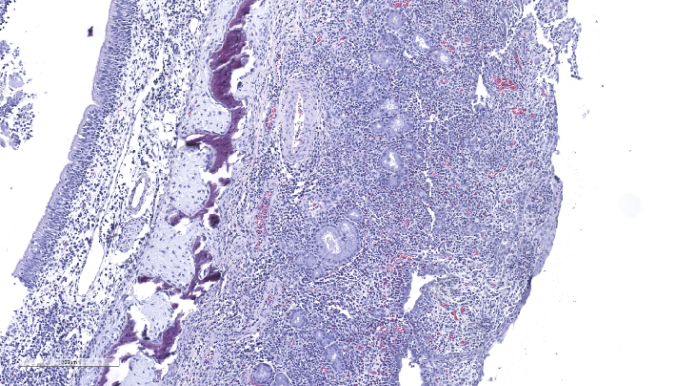

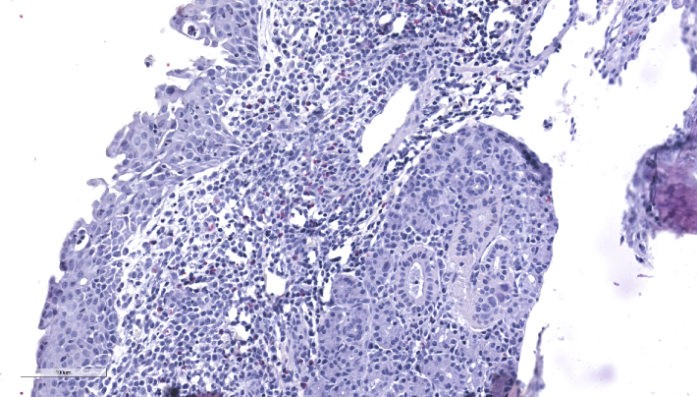


b. d. f.

IHC


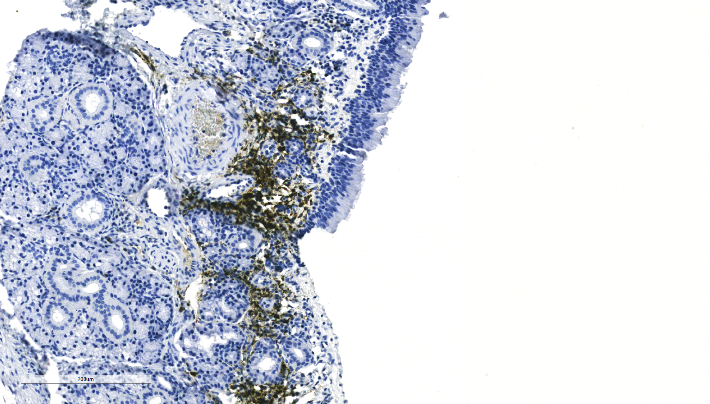

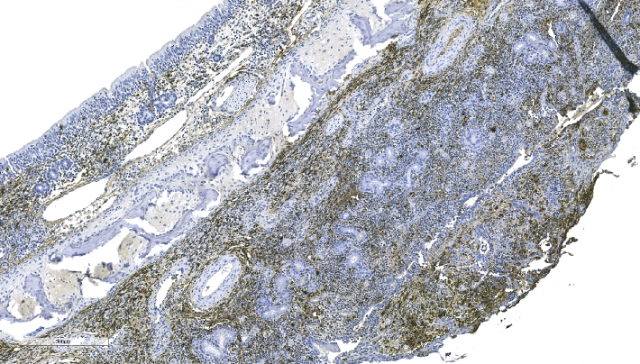

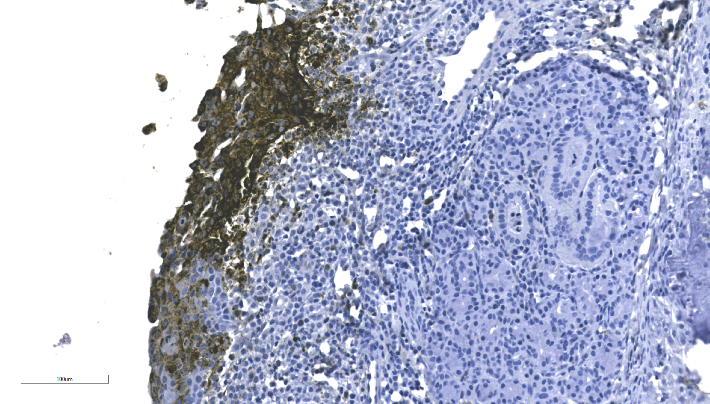


**Supplement Figure 7: Histopathology and immunohistochemistry findings in nasal turbinates of Reston virus-infected pigs.** At four days post-infection (dpi), both animals necropsied had mild to moderate submucosal inflammation noted in the nasal turbinates (a) including the presence of viral antigen detected by immunohistochemistry IHC) (b). These findings were also present in the animals necropsied at six dpi (c, d). Inflammation was also noted in the nasal turbinates of both animals at seven dpi (e) and viral antigen was detected in both the submucosa and epithelium (f). Tissues originated from Pig 8 (a,b), Pig 10 (c,d), and Pig 12 (e,f).

4 dpi 6 dpi 7 dpi

a. c. e.

HE


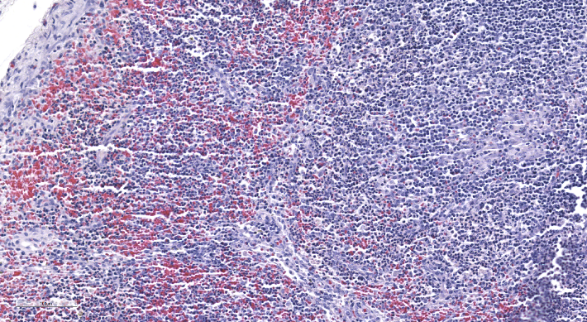

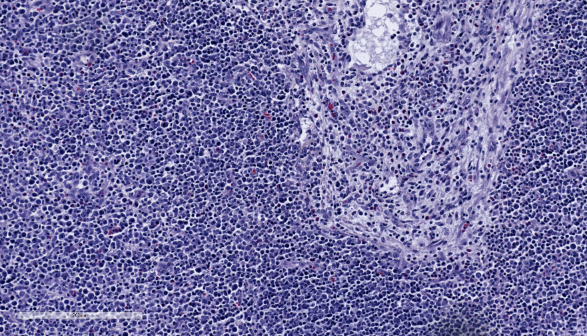

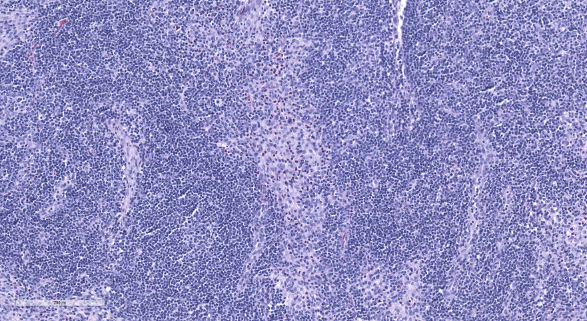


b. d. f.

IHC


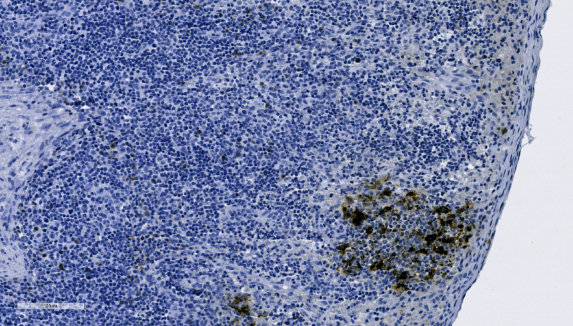

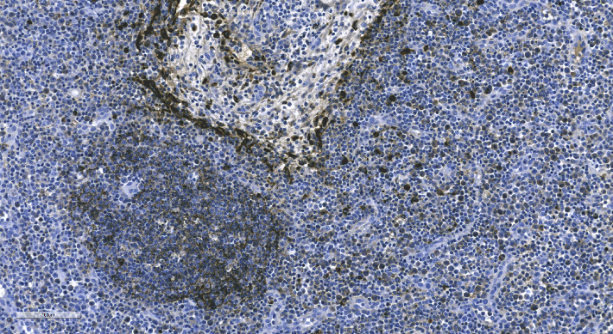

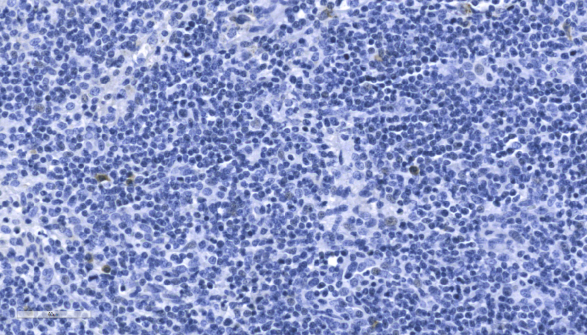


g. h. i.

IHC


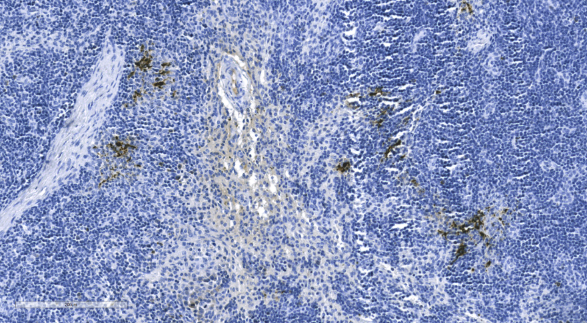

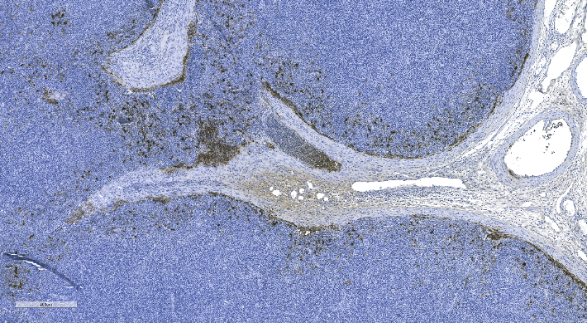

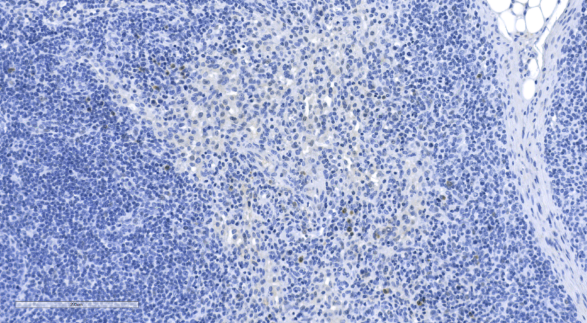


**Supplement Figure 8: Histopathology and immunohistochemistry findings in lymph nodes of RESTV-infected pigs.** At four days post-infection (dpi) (a), there was necrosis, hemorrhage, and neutrophil infiltration observed in the tracheobronchial lymph node which was associated with the presence of viral antigen (b) detectable by immunohistochemistry (IHC). At six dpi, there was an increased number of lymphocytes with pyknotic nuclei (c), suggestive of either lymphocyte necrosis or apoptosis, in the tracheobronchial lymph node, as well as a large amount of viral antigen detected by IHC in both the tracheobronchial (d). Lesions were not noted at seven dpi (e), but viral antigen was detected by IHC in a few scattered cells (some noted with arrows) (f). No histopathologic lesions were detected in the mandibular lymph node (HE examples not shown), but varying levels of viral antigen was detected by IHC, including localized, prominent staining at four dpi (g), extensive staining at six dpi (h), and staining in only a few, scattered cells (some noted with arrows) at seven dpi (i). Tissues originated from Pig 9 (a, b, g), Pig 10 (c, d, h), Pig 13 (e, f), and Pig 12 (i).


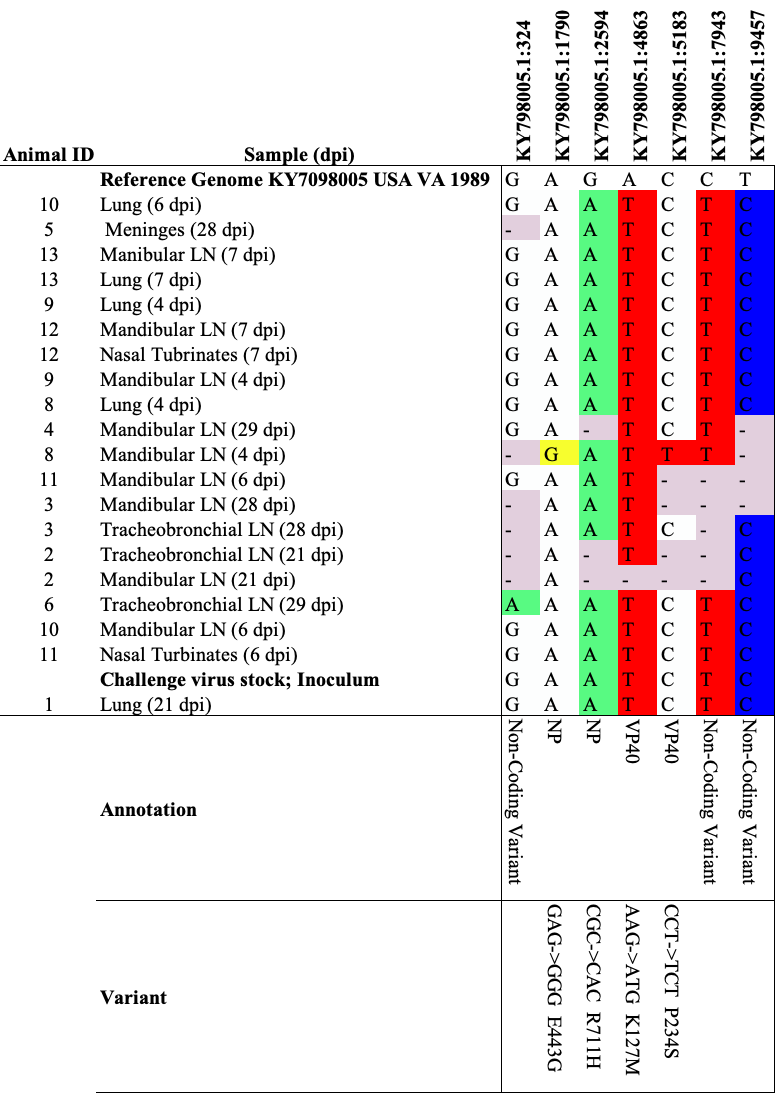


**Supplement Figure 9: Comparison of virus sequences from terminal tissue samples in Reston virus infected pigs confirms that infection of pigs did not require species-specific adaptation.** Whole genome sequencing was used to determine the sequence of virus used as the inoculum, as well as virus recovered from tissues collected from each of the study animals. These sequences were compared to the annotated reference genome for isolate USA_VA_1989. As shown on this table, a total of seven single nucleotide polymorphisms (SNPs) were noted with three being found in non-coding regions and four being found in the coding regions for the virus nucleoprotein (NP) and viral protein 40 (VP40). Dashes indicate regions were sequence coverage was low. LN, lymph node; dpi, day post-inoculation).
